# Supplementary material for: Non-pharmacological fatigue interventions for patients with a primary brain tumour: a scoping review protocol
Source: BMJ Open. 2023 Dec 14;13(12):e078183. doi: 10.1136/bmjopen-2023-078183 (PMC10729204; doi:10.1136/bmjopen-2023-078183)
Supplement: Supplementary data [file bmjopen-2023-078183supp001.pdf]

Supplementary Information

Supplementary Information 1: MEDLINE (Ovid) search strategy

1. exp brain neoplasms/
2. exp glioma/
3. exp neoplasms/
4. brain cancer.mp. or glioma.mp. or tumor.mp. or neurooncology.mp. or neoplasm.mp. or lump.mp. or anaplastic astrocytoma.mp. or glioblastoma.mp. or medulloblastoma.mp. or ependymoma.mp. or oligodendroglioma.mp.
5. 1 or 2 or 3 or 4
6. exp fatigue/
7. (cancer related fatigue or tiredness or exhaustion or weariness or lethargy or lassitude or drained or phasic alertness or executive function or sleep).mp.
8. 6 or 7
9. exp nonpharmacologic therapy/
10. (nonpharmacologic or holistic or nonmedical or unmedicated or nonpharmaceutical).mp.
11. 9 or 10
12. exp treatment outcome/
13. (treatment or program or intervention or therapy or therapeutics or care or ministrations or psychosocial or exercise or physical activity or rehabilitation or cognitive or measure or measurements or scale or education or information or instruments).mp.
14. 12 or 13
15. exp quality of life/
16. (welfare or wellbeing or happiness or quality of life or standard of living or psychooncology or survivorship or mental health).mp.
17. 15 or 16
18. 5 or 8 or 11 or 14 or 17
19. animals not humans.sh.
20. exp child/
21. 19 not 20
22. limit 21 to english language
23. limit 22 to ("2006/01/01"-"current time")

Exp = exploded indexing term, mp = Multifield plus, sh = Subject heading.

Supplementary Information 2: EMBASE (Ovid) search strategy

1. Exp Brain Neoplasms/
2. Exp Glioma/
3. Exp Neoplasms/
4. (Brain cancer OR Glioma OR tumor OR neuro-oncology OR neoplasm OR lump OR anaplastic astrocytoma OR glioblastoma OR medulloblastoma OR ependymoma OR oligodendroglioma).tw.
5. Neurooncology/
6. 1 or 2 or 3 or 4 or 5
7. Exp Fatigue/
8. (Cancer related fatigue OR tiredness OR exhaustion OR weariness OR lethargy OR lassitude OR drained OR phasic alertness OR executive function OR sleep).tw.
9. Exp non-pharmacological therapy/
10. (non-pharmacological OR holistic OR non-medical OR unmedicated OR non-pharmaceutical).tw.
11. Exp Treatment/
12. (program OR intervention OR therapy OR therapeutics OR care OR ministrations OR psychosocial OR exercise OR physical activity OR rehabilitation OR cognitive OR measure OR measurements OR scale OR education OR information OR instruments).tw.
13. Exp Quality of Life/
14. (welfare OR wellbeing OR happiness OR quality of life OR standard of living OR psycho-oncology OR survivorship OR mental health).tw.
15. 6 or 8 or 10 or 12 or 14
16. Exp Animals/ not human/
17. Exp Child/
18. 16 not 17
19. Limit 18 to English language
20. Limit 19 to 2006-current

Exp = exploded indexing term, tw = text word search in title or abstract fields.

Supplementary Information 3: CENTRAL (*The Cochrane Library*) search strategy

1. MeSH descriptor: [Brain Neoplasms] explode all trees
2. MeSH descriptor: [Glioma] explode all trees
3. MeSH descriptor: [Neoplasms]
4. Brain cancer OR glioma OR tumor OR neuro-oncology OR neoplasm OR lump OR anaplastic astrocytoma OR glioblastoma OR medulloblastoma OR ependymoma OR oligodendroglioma
5. MeSH descriptor: [Neuro-oncology] explode all trees
6. 1 or 2 or 3 or 4 or 5
7. MeSH descriptor: [Fatigue] explode all trees
8. Cancer-related fatigue or tiredness or exhaustion or weariness or lethargy or lassitude or drained or phasic alertness or executive function or sleep
9. 7 or 8
10. MeSH descriptor: [Complementary Therapies] explode all trees
11. Non-pharmacological or holistic or non-medical or unmedicated or non-pharmaceutical
12. 10 or 11
13. MeSH descriptor: [Therapeutics] explode all trees
14. Treatment or program or intervention or therapy or therapeutics or care or ministrations or psychosocial or exercise or physical activity or rehabilitation or cognitive or measure or measurements or scale or education or information or instruments
15. 13 or 14
16. MeSH descriptor: [Quality of Life] explode all trees
17. Welfare or wellbeing or happiness or quality of life or standard of living or psycho-oncology or survivorship or mental health
18. 16 or 17
19. 6 or 9 or 12 or 15 or 18
20. MeSH descriptor: [Animals] explode all trees, except humans
21. MeSH descriptor: [Child] explode all trees
22. 20 or 21
23. 19 not 22
24. Limit 23 to 2006-current
25. Limit 24 to English

Supplementary Information 4: CINAHL (EBSCO*Host*) search strategy

1. (Brain tumor).mh
2. (Glioma).mh
3. (Neoplasms).mh
4. Brain cancer OR Glioma OR tumor OR neuro oncology OR neoplasm OR lump OR anaplastic astrocytoma OR glioblastoma OR Medulloblastoma OR Ependymoma OR Oligodendroglioma
5. MeSH descriptor: [Neurooncology] explode all trees
6. 1 or 2 or 3 or 4 or 5
7. (fatigue).mh
8. Cancer related fatigue or tiredness or exhaustion or weariness or lethargy or lassitude or drained or phasic alertness or executive function or sleep
9. 7 or 8
10. (non-pharmacological).mh
11. non-pharmacological or holistic or non-medical or unmedicated or non-pharmaceutical
12. 10 or 11
13. (intervention).mh
14. Treatment or program or intervention or therapy or therapeutics or care or ministrations or psychosocial or exercise or physical activity or rehabilitation or cognitive or measure or measurements or scale or education or information or instruments
15. 13 or 14
16. (quality of life).mh
17. Welfare or wellbeing or happiness or quality of life or standard of living or psycho-oncology or survivorship* or mental health
18. 6 or 9 or 12 or 15 or 17
19. animals/ not humans
20. children
21. 19 Not 20
22. Limit 21 to 2006- current
23. Limit 22 to English

*mh: Major heading, mm: Minor heading. *: truncation*

Supplementary Information 5: SCOPUS search strategy

1. EXP Brain tumor$
2. EXP Glioma$
3. EXP Neoplasms$
4. Brain cancer OR Glioma OR tumor OR neuro oncology OR neoplasm OR lump OR anaplastic astrocytoma OR glioblastoma OR Medulloblastoma OR Ependymoma OR Oligodendroglioma
5. EXP Neurooncology$
6. 1 OR 2 OR 3 OR 4 OR 5
7. EXP Fatigue$.TW.
8. "Cancer related fatigue" OR tiredness OR exhaustion OR weariness OR lethargy OR lassitude OR drained OR "phasic alertness" OR "executive function" OR sleep
9. 7 OR 8
10. EXP non-pharmacological$
11. non-pharmacological OR holistic OR "non-medical" OR unmedicated OR "non-pharmaceutical"
12. 10 OR 11
13. EXP intervention$
14. Treatment OR program OR intervention OR therapy OR therapeutics OR care OR ministrations OR psychosocial OR exercise OR "physical activity" OR rehabilitation OR Cognitive OR measure OR measurements OR scale OR education OR information
15. 13 OR 14
16. EXP Quality of Life$
17. Welfare OR wellbeing OR happiness OR "quality of life" OR "standard of living" OR "psycho-oncology" OR survivorship$ OR "mental health"
18. 16 OR 17
19. 6 OR 9 OR 12 OR 15 OR 18
20. EXP animals/ NOT humans.SH.
21. EXP children#.SH.
22. 20 OR 21
23. 19 NOT 22
24. LIMIT-TO (SRCTITLE, "SCOPUS") AND PUBYEAR > 2005
25. LIMIT-TO (LANGUAGE, "English")

$ = identify all words beginning with the stem, # = controlled vocabulary alternative spellings, Exp = exploded indexing term, TW = text word search in title or abstract fields.

Supplementary Information 6: Psych info (EBSCO*host*) search strategy

1. "Brain tumour".sh
2. "Glioma".sh
3. "Neoplasms".sh
4. Brain cancer OR Glioma OR tumor OR neuro-oncology OR neoplasm OR lump OR anaplastic astrocytoma OR glioblastoma OR medulloblastoma OR Ependymoma OR Oligodendroglioma
5. "Neurooncology".sh
6. 1 or 2 or 3 or 4 or 5
7. "Fatigue".tw
8. Cancer related fatigue or tiredness or exhaustion or weariness or lethargy or lassitude or drained or phasic alertness or executive function or sleep
9. 7 or 8
10. "Non-pharmacological".tw
11. non-pharmacological or holistic or non-medical or unmedicated or non-pharmaceutical
12. 10 or 11
13. "Intervention".tw
14. Treatment or program or intervention or therapy or therapeutics or care or ministrations or psychosocial or exercise or physical activity or rehabilitation or cognitive or measure or measurements or scale or education or information or instruments
15. 13 or 14
16. "Quality of Life".tw
17. Welfare or wellbeing or happiness or quality of life or standard of living or psycho-oncology or survivorship or mental health
18. 16 or 17
19. 6 or 9 or 12 or 15 or 18
20. "Animals".sh NOT Humans.sh
21. "Child".sh
22. 20 or 21
23. 19 NOT 22
24. Limit 23 to English
25. Limit 24 to United Kingdom
26. Limit 25 to 2006-Current

Sh = Subject heading, tw = text word search in title or abstract fields.

Supplementary Information 7: AMED (EBSCO*host*) search strategy

1. exp Brain Neoplasms/
2. exp Glioma/
3. exp Neoplasms/
4. brain cancer OR glioma OR tumor OR neuro-oncology OR neoplasm OR lump OR anaplastic astrocytoma OR glioblastoma OR medulloblastoma OR ependymoma OR oligodendroglioma
5. exp Neurooncology/
6. (1 or 2 or 3 or 4 or 5)
7. exp Fatigue/ or Fatigue.tw.
8. cancer related fatigue or tiredness or exhaustion or weariness or lethargy or lassitude or drained or phasic alertness or executive function or sleep
9. (7 or 8)
10. exp Complementary Therapies/ or exp Holistic Health/
11. non-pharmacological or holistic or non-medical or unmedicated or non-pharmaceutical
12. (10 or 11)
13. exp Treatment Outcome/ or exp Rehabilitation/ or exp Psychosocial Intervention/ or exp Exercise/ or exp Cognitive Therapy/ or exp measure/ or exp measurements/ or exp scale/ or exp education/ or exp information/ or exp instruments
14. treatment or program or intervention or therapy or therapeutics or care or ministrations or psychosocial or exercise or physical activity or rehabilitation or cognitive
15. (13 or 14)
16. exp Quality of Life/
17. welfare or wellbeing or happiness or quality of life or standard of living or psycho-oncology or survivorship or mental health
18. (16 or 17)
19. animals/ not humans.sh.
20. children.sh.
21. (19 not 20)
22. limit 21 to english language
23. limit 22 to "2006-Current"

Exp = exploded indexing term, tw = text word search in title or abstract fields, Sh = Subject heading.

Supplementary Information 8: ClinicalTrials.gov search strategy

1. (Brain cancer OR Glioma OR tumor OR tumor OR neuro oncology OR neoplasm OR lump OR anaplastic astrocytoma OR glioblastoma OR Medulloblastoma OR Ependymoma OR Oligodendroglioma AND (fatigue OR Cancer related fatigue OR tiredness OR exhaustion or weariness OR lethargy OR lassitude OR drained OR phasic alertness OR executive function OR sleep) AND (non-pharmacological OR holistic OR non-medical OR unmedicated OR non-pharmaceutical) AND (Intervention OR Treatment OR program OR intervention OR therapy OR therapeutics OR care or ministrations OR psychosocial OR exercise OR physical activity OR rehabilitation OR Cognitive OR measure OR measurements OR scale OR education OR information OR) AND (Quality of Life OR Welfare OR wellbeing OR happiness OR quality of life OR standard of living OR psycho-oncology OR survivorship OR mental health)

Boolean search terms: AND, OR, NOT

Supplementary Information 9: Scoping Review Search Strategy overview

**MesH**	**Concept 1: Brain tumor** *(Primary/non-metastatic)*	**Concept 2: Fatigue**	**Concept 3: Non-Pharmacological**	**Concept 4: Interventions**	**Concept 5: Quality of life**
**Mesh Key search terms**	Brain cancer, glioma, tumor, tumor, neuro oncology, neoplasm, lump, anaplastic astrocytoma, glioblastoma, Medulloblastomas, Ependymomas, Oligodendrogliomas	Fatigue, cancer related fatigue, tiredness, exhaustion, weariness, lethargy, lassitude, drained, phasic alertness, executive function, sleep	Non-pharmacological, holistic, non-medical, unmedicated, non-pharmaceutical	Treatment, program, intervention, therapy, therapeutics, care, ministrations, psychosocial, exercise, physical activity, Cognitive, rehabilitation, measure, measurements, scale, education, information, instruments	Welfare, wellbeing, happiness, quality of life, standard of living, psycho-oncology, survivorship, mental health
**Data bases** *Searching electronic databases, conferences and grey literature.*	• MEDLINE, EMBASE, CINAHL, CENTRAL, SCOPUS, Psych info, AMED.<br>• MEDLINE – IN PROCESS and Other Non-Indexed Citations Via OVID.<br>• Relevant conference proceedings will be searched by the lead author using the OVID, PROSPERO, The Cochrane Library, SCOPUS and the International Clinical Trials Registry platforms.<br>• Grey literature database ETHOS will also be searched.				
**Time frame**	2006- 2023.				

Supplementary Information 10: PICO model for Scoping Review on Non-Pharmacological Interventions for Fatigue in Primary Brain Tumor Patients

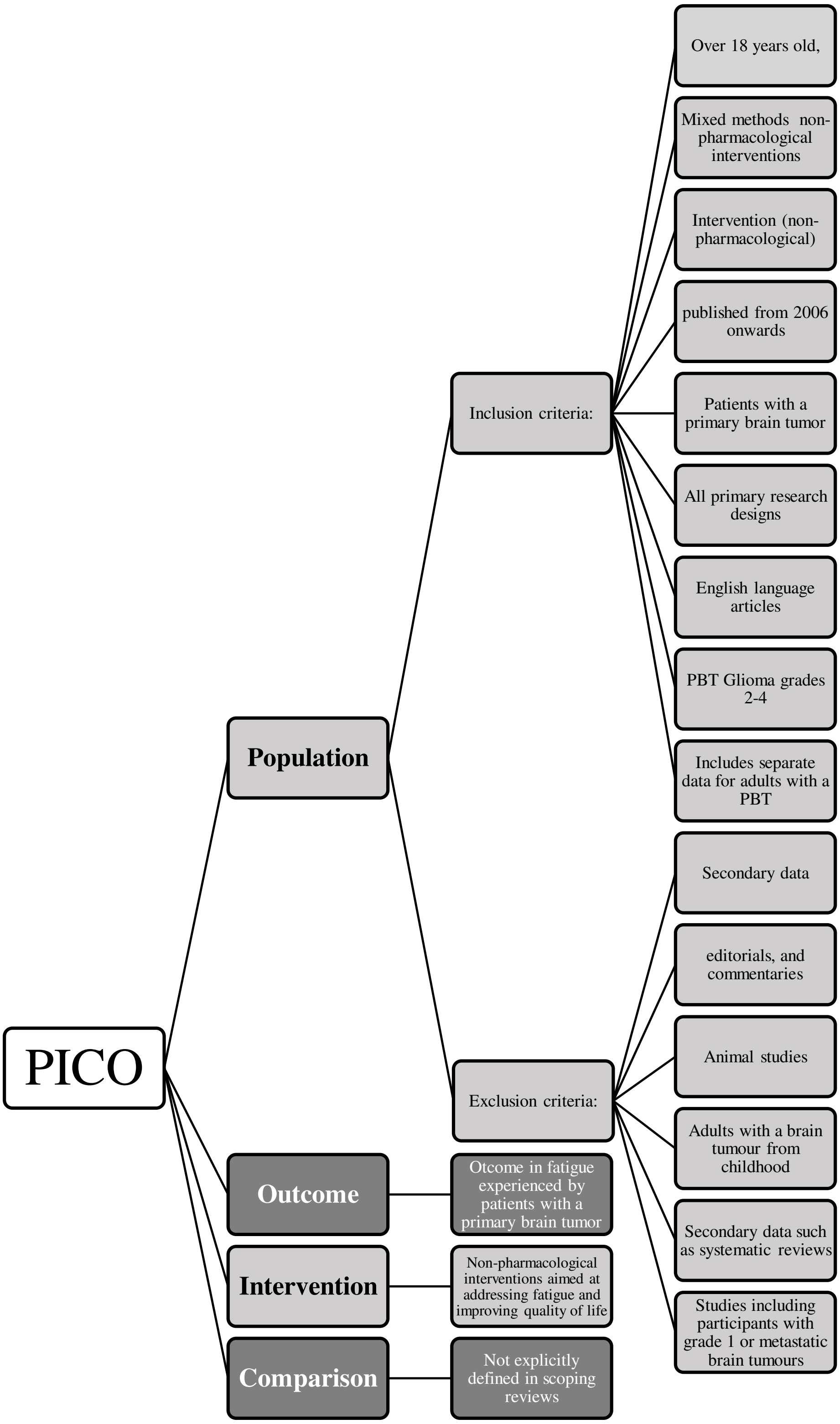

Supplementary Information 11: Data charting Form

	Study number	1	2	3	4
Study information	Author				
	Title				
	Publication year				
	Study method				
	Sample size (participants, arms, control)				
	Study location (geographic or country region)				
	Study setting (hospital, community centre, charity center, home)				
	Main conclusion				
Participant demographics	Age (mean, standard deviation, arm ages)				
	Gender				
	Population description (ethnicity, socio demographic)				
	Tumour type and grade				
	Comorbidities				
Study findings	Study Duration (intervention length &timepoints/ results)				
	Intervention type				
	Intervention provider (medical professionals, allied health professionals, laypersons or trained volunteers, patients' family members or caregivers, and Others- description)				
	Intervention provider training				
	Primary outcome				
	baseline data outcomes				
	Study findings				
	secondary outcomes				
	follow up data timepoints				
	Author recommendations				
	Personal reflections				
