# Supplementary material for: Non-pharmacological fatigue interventions for patients with a primary brain tumour: a scoping review protocol
Source: BMJ Open. 2023 Dec 14;13(12):e078183. doi: 10.1136/bmjopen-2023-078183 (PMC10729204; doi:10.1136/bmjopen-2023-078183)
Supplement: Author's manuscript [file bmjopen-2023-078183.draft_revisions.pdf]

BMJ Open is committed to open peer review. As part of this commitment we make the peer review history of every article we publish publicly available.

When an article is published we post the peer reviewers' comments and the authors' responses online. We also post the versions of the paper that were used during peer review. These are the versions that the peer review comments apply to.

The versions of the paper that follow are the versions that were submitted during the peer review process. They are not the versions of record or the final published versions. They should not be cited or distributed as the published version of this manuscript.

BMJ Open is an open access journal and the full, final, typeset and author-corrected version of record of the manuscript is available on our site with no access controls, subscription charges or pay-per-view fees (http://bmjopen.bmj.com).

If you have any questions on BMJ Open's open peer review process please email info.bmjopen@bmj.com

BMJ Open

# Non-pharmacological fatigue interventions for patients with a primary brain tumour: a scoping review protocol.


| | |
|---|---|
| Journal: | *BMJ Open* |
| Manuscript ID | bmjopen-2023-078183 |
| Article Type: | Protocol |
| Date Submitted by the Author: | 26-Jul-2023 |


| | |
|---|---|
| Complete List of Authors: | Simms-Moore, Rachael; University of the West of England, School of Health and Social Wellbeing<br>Dures, Emma; University of the West of England, Rheumatology and Self-Management<br>Barua, Neil; Southmead Hospital, Neurosurgery<br>Cramp, Fiona; University of the West of England, College of Health, Science & Society |


| | |
|---|---|
| Keywords: | Fatigue, Head & neck tumours < ONCOLOGY, PUBLIC HEALTH |


SCHOLARONE™
Manuscripts

# **TITLE** Non-pharmacological fatigue interventions for patients with a primary brain tumour: a scoping review protocol.

**Word count**: 2,468

Rachael Simms-Moore[1], Dr Emma Dures[2], Dr Neil Barua[3], Professor Fiona Cramp[4].

[1]Rachael Simms-Moore, University of the West of England, Bristol, rachael.simms-moore@uwe.ac.uk
[2]Dr Emma Dures, University of the West of England, Bristol, Emma2.Dures@uwe.ac.uk
[3]Dr Neil Barua, North Bristol NHS Trust, neilbarua@doctors.org.uk
[4]Professor Fiona Cramp, University of the West of England, Bristol, Fiona.Cramp@uwe.ac.uk

**Corresponding author:**
Rachael Simms-Moore[1]

Postal address:
School of Health and Social Wellbeing
College of Health, Science and Society
UWE Bristol
Glenside Campus
Blackberry Hill
BS16 1DD

Email address: rachael.simms-moore@uwe.ac.uk

Telephone: 07534389777

For peer review only



For peer review only - http://bmjopen.bmj.com/site/about/guidelines.xhtml

## ABSTRACT

### Introduction

Fatigue is the most prevalent symptom for patients with a primary brain tumour. Significantly reducing quality of life and limiting daily activities. Currently, there are limited options for managing cancer related fatigue in patients with a primary brain tumour, using non-pharmacological methods. The objective of this scoping review is to understand current and emerging extent evidence in relation to non-pharmacological cancer related fatigue interventions for patients with a primary brain tumour.

### Methods and analysis

Electronic databases OVID and EBSCO platforms: MEDLINE, EMBASE, and CINAHL will be searched. In addition, PROSPERO, The Cochrane Library, and ISI Web of Science will also be searched. Trials registries CENTRAL and the International Clinical Trials Registry platform will also be searched for ongoing research. Inclusion criteria: studies from 2006 onwards, primary research on non-pharmacological interventions in patients with a primary brain tumour (>18 years). A PRISMA flow diagram to summarise the screening process and results.

Quantitative data will be analysed descriptively, while content analysis will be used for qualitative data.

Findings will map the existing and emerging evidence on non-pharmacological interventions for cancer-related fatigue in patients with primary brain tumours.

This will provide insights into the extent and nature of the evidence in this evolving field, identifying gaps in knowledge and research priorities, and guide further investigations in this area.

### Ethics and dissemination

Ethical approval is not required for this scoping review. Findings will be disseminated via relevant peer-reviewed journals, PhD thesis, conference presentations, and shared with relevant charities and health professionals.

**Keywords:** primary brain tumour, fatigue, adult, cancer related fatigue, non-pharmacological

## ARTICLE SUMMARY

### Strengths and limitations of this study

- Using the PRISMA- Extension for Scoping Reviews framework for replicability and transparency.
- Two independent researchers will assess included studies using a validated quality assessment tool.
- Includes all primary research published since 2006.
- Bias in studies that do not have a control comparison.
- Captures a broader range of evidence in an under researched problem in a relatively small patient population.



For peer review only

For peer review only - http://bmjopen.bmj.com/site/about/guidelines.xhtml

## INTRODUCTION

A primary brain tumour (PBT) is a growth of cells originating in the brain that multiplies in "an abnormal, uncontrollable way" [1]. PBT's can be categorised into high grade (grades 3-4) and lower grade (grade 1-2) tumours, with high-grade PBTs exhibiting more rapid growth than lower-grade PBT's [1]. Gliomas are the most common type of PBT, arising from glial cells in the brain and accounting for over half of PBT's [2].

Fatigue is the most prevalent symptom for patients with PBT [3]. Fatigue associated with having cancer and consequent treatments such as radiotherapy and chemotherapy is known as Cancer-Related Fatigue (CRF) [4]. The National Comprehensive Cancer Network (NCCN) defines CRF as "a distressing, persistent, subjective sense of physical, emotional, and/or cognitive tiredness or exhaustion related to cancer or cancer treatment that is not proportional to recent activity and interferes with usual functioning" [5]. CRF can involve mental, physical, and/or psychological components, with variability between patients and over time [6]. Along with experiencing unusually elevated levels of fatigue disproportionate to activity, CRF cannot be mitigated through sleep or rest [7]. CRF can significantly reduce cancer patients' quality of life [8], limiting their ability to work, continue treatment, socialise, or engage in daily activities [9-11]. Indeed, a study found that patients felt that CRF negatively impacted daily life more than pain [4].

There are two potential avenues for treating CRF, pharmacological interventions and non-pharmacological interventions. Non-pharmacological interventions are defined as interventions where pharmaceutical medications are not used as treatment [12], for example, lifestyle changes, psychosocial therapies, and physical activity.

Evidence suggests that patients do not want to take more drugs, with approximately 50% of patients with chronic illness not taking their medications as prescribed [13,14]. A Cochrane review (updated 2022), of "Interventions for the management of CRF in adults with a primary brain tumour" explored efficacy of interventions. This review included three RCTs that had investigated pharmacological interventions for CRF [15]. All three medicines studied, Modafinil, Dexamfetamine sulphate and Amodofinil were psychostimulants; based upon the theory that promoting wakefulness could reduce CRF in patients with PBT. All three studies reported no significant effect of the pharmacological intervention on CRF compared with the control arm [16-18].

Currently, there are limited options for managing CRF in patients with a PBT [15]. Non-pharmacological evidence to date has predominantly focussed upon movement interventions, physical activity and exercise. Although the terms have been used interchangeably [19], they differ. Exercise interventions being structured, planned, repetitive activities aimed at improving fitness and health outcomes and physical activity interventions covering a broader range of movement and aim to increase overall activity levels for health and well-being [20].

In a recent systematic review of physical activity and exercise in adults with primary brain cancer, two randomised controlled trials (RCT) reported improvements in patient fatigue [21].

One pilot RCT focused on an exercise program to improve cognitive function in patients with a PBT. It demonstrated small- to medium self-reported improvements in CRF, specifically in the areas of physical fatigue and reduced activity, with a medium effect compared to the control arm [22].

Another study by Milbury et al (2019) explored the effectiveness of dyadic yoga as an intervention for glioma patients and their families. Their study demonstrated a small



For peer review only - http://bmjopen.bmj.com/site/about/guidelines.xhtml

clinically significant difference between the control and intervention groups, favouring the dyadic yoga intervention [23].

Overall, these findings highlight the potential of physical activity interventions in reducing CRF in patients with a PBT.

There is some limited research indicating that patients with PBT may benefit from cognitive rehabilitation programs [21] or Health Coaching programs [24]. Although not specifically studied in patients with a PBT, cognitive behavioural therapy (CBT) has been shown to reduce severe CRF in a variety of cancers [25, 26]. CBT is a talking therapy that helps treat mental health problems by focusing on how thoughts, beliefs, and attitudes impact emotions and behaviours (27). CBT teaches coping skills and challenges negative thinking patterns, aiming to improve emotional well-being (27).

Within the Cochrane review, non-pharmacological studies were organised into four main categories: educational programmes, physical activity, psychosocial/ talking therapies, and cognitive rehabilitation programs. Overall findings indicated that there was insufficient evidence to support the effectiveness and safety of non-pharmacological treatments for CRF in patients with PBT [15].

Beneficial effects of non-pharmacological interventions have been shown for (CRF), but the evidence to date in patients with a PBT is limited, with few RCTs specific to this population. Furthermore, pharmacological interventions have demonstrated limited benefits in addressing CRF and patients are often hesitant to add more medications to their regimen.

**Review aims**

This protocol outlines the methodology for a scoping review to map both current and emerging evidence regarding non-pharmacological interventions for cancer-related fatigue in patients with primary brain tumours. By incorporating all primary research evidence, the review has the potential to capture a wide range of interventions. The overall aim is to provide a comprehensive overview of the extent and type of evidence available in this developing field, while also identifying gaps and priorities for future research. Its ultimate goal is to contribute to a better understanding of the current state of knowledge in this area.

## METHODS AND ANALYSIS

**Study design**

Scoping reviews can be used to explore a research area that is developing [28] and has not been reviewed comprehensively [29]. Including a broader range of methods compared to a systematic or Cochrane review [15], a scoping review will capture research findings beyond RCTs. This review will utilise the PRISMA Extension for Scoping Reviews (PRISMA-ScR) framework (see additional file, research checklist) [28].

**Study question**

The review will address the question: What interventions have addressed the management of CRF in patients with PBT? The review aims to evaluate the quality of the evidence and study outcomes to determine if any recommendations can be made for clinical practice. Additionally, the research priorities for further investigation will be identified.

**Identifying relevant studies**

The scoping review will include all primary research designs published from 2006 onwards. This date corresponds with the National Institute for Health and Clinical Excellence (NICE) introducing updated guidance "Improving Outcomes for People with Brain and Other CNS Tumours" [30]. This guidance promoted multidisciplinary care for patients during treatment, in recovery and during palliative stages due to patients varying in their care requirements [30].



For peer review only - http://bmjopen.bmj.com/site/about/guidelines.xhtml

A search strategy has been developed with input from a university-based specialist subject librarian and modified for each database (see Supplementary Information 3-8). The Peer Review of Electronic Search Strategy checklist will be used to validate the search strategy [31].

To ensure a comprehensive literature search, a range of generalised and specialised electronic databases will be searched [32, 33]. Details of the keyword searches are presented in supplementary information 3-10. The following electronic databases will be searched using the OVID and EBSCO platforms: MEDLINE, EMBASE, and CINAHL. In addition, PROSPERO, The Cochrane Library, and ISI Web of Science will also be searched. Trials registries CENTRAL and the International Clinical Trials Registry platform will also be searched for ongoing research.

The databases will be searched using medical subject headings (MeSH) and key search terms (see Supplementary Information 2). Keywords for each subject heading were developed using a mix of the Yale MeSH analyzer software [34], free-text terms and explode command in MEDLINE, thesaurus recommendations and preferred vocabulary across databases.

In-process research such as MEDLINE – IN PROCESS and Other Non-Indexed Citations will be searched via OVID to minimise publication bias. Grey literature database Ethos will also be searched to minimize bias as positive findings are more likely to be published within peer reviewed journals [35].

The lead author (RSM) will upload all studies and articles into referencing software EndNote-20 [36] and utilize scoping review software Covidence [37] to store the data. Any duplicates will be removed at this stage.

**Selection of studies**

Studies found will then be screened using inclusion and exclusion criteria (Table 1).

Table 1: Inclusion and exclusion criteria for studies

| **Inclusion** | **Exclusion** |
|---|---|
| • Any primary research designs<br>• Published from year 2006 onwards<br>• Available in English language<br>• Includes separate data for adults with a PBT<br>• PBT Glioma grades 2-4<br>• Studies including mixed non-pharmacological interventions<br>• Non-pharmacological intervention being investigated for its effect on CRF | • Animal studies<br>• Children (Persons under 18 years)<br>• Editorial or commentaries<br>• Secondary data such as systematic reviews<br>• Studies including participants with grade 1 or metastatic brain tumours |

In this study PBT's of grade 2-4 will be included. This is supported by previous research by Röttgering (2022), finding that there was not a significant difference in fatigue severity reported by individuals with different tumour grades ranging from 2-4 [3].

The screening process will begin with two independent reviewers (RSM and AB) reviewing the titles, followed by the abstracts, and any resources with doubt or disagreement will be retained. Next, both reviewers will independently screen the full texts using the inclusion and exclusion criteria. If there are any disagreements these will be discussed initially and if necessary, the director of studies (FC) will be consulted for conciliation.

**Data charting process**

Study characteristics, design, aims, participants, interventions, outcome measures, findings and authors conclusions will be collected into a data charting form (DCF) (see



For peer review only - http://bmjopen.bmj.com/site/about/guidelines.xhtml

Supplementary Information 1). The DCF will initially be tested by the lead author and first supervisor (RSM and FC) and refined as necessary. An independent reviewer (AB) will carry out data extraction on the studies for process validation.

**Analysis of data**

Data analysis is likely to be narrative with content analysis considered for qualitative data. All data will be summarised to produce a map of the current literature available.

**Assessment of methodological quality of individual studies**

Two reviewers (AB and RSM) will independently complete a quality assessment of each included study using the enhanced mixed methods appraisal tool (MMAT). The MMAT tool was selected as the review is likely to contain quantitative and qualitative studies, therefore a mixed methods quality assessment tool is appropriate [38]. The enhanced MMAT version was selected as it focuses on five core quality criteria to increase efficiency [39].

**Collating, summarising, and reporting**

Gaps will be highlighted and where appropriate recommendations for future research and clinical practice will be identified. Findings will be presented in a PRISMA flow diagram, mapping the number of resources identified and reasons for those included and excluded [28]. Included studies will be summarised in tabular format.

Further figures may be used to illustrate a map of the literature and gaps highlighted.

**Patient and public involvement**

This research has been discussed with patient research partners and representatives of two relevant brain tumour charities who agree that this is a priority for research. They will continue to work alongside the lead researcher in an advisory role and the findings from the review will be shared with them for further discussion.

The findings derived from this study have the potential to inform and guide the research team in their endeavour to design a non-pharmacological intervention for managing fatigue in patients with a brain tumour.

**Discussion**

There is no universally accepted standard treatment for CRF, and a broad range of diverse non-pharmacological strategies exist for managing fatigue in patients with a PBT [15]. This scoping review aims to expand upon the findings of a previous Cochrane review and comprehensively examine the available evidence encompassing a broad range of non-pharmacological interventions for CRF in patients with PBT.

By incorporating all primary research evidence, the review seeks to provide a comprehensive overview of the extent and type of evidence available in this developing field. It aims to identify gaps and priorities for future research, ultimately contributing to a better understanding of the current state of knowledge in this area. The results may identify interventions that are worthy of further research investment and provide recommendations for clinical practice. Through this exploration of non-pharmacological interventions for managing CRF in patients with PBT, we aim to enhance understanding and guide decision-making and identify potential interventions that could benefit patients with a PBT in the future.

**ETHICS AND DISSEMINATION**



For peer review only - http://bmjopen.bmj.com/site/about/guidelines.xhtml

Ethical approval is not required for this scoping review. The findings of this review will be disseminated via relevant peer-reviewed journals, PhD thesis, conference presentations, and through sharing findings with relevant charities and health professionals.

**Amendments**

The protocol will be closely followed throughout with RSM regularly reporting to the supervisory team (FC, NB & ED). If any amendments are made to the published study protocol, these will be reported in the final publication.

**Supplementary Information**

Search strategies for databases

**Abbreviations**

CRF- Cancer related fatigue
PBT- primary brain tumour
RSM- Rachael Simms-Moore (Lead Author)
FC- Fiona Cramp (Director of Studies)
NB- Neil Barua (Supervisor)
ED- Emma Dures (Supervisor)
AB- Amy Belisario (independent reviewer)

**Acknowledgements**

None

**Funding**

This scoping review is funded as part of a PhD studentship by UWE Bristol and Somerset, Wiltshire, Avon and Gloucestershire (SWAG) Cancer Services held by RSM. The funders had no role in this scoping review protocol.

**Availability of data and materials**

The datasets used and/or analysed during the current study are available from the corresponding author on reasonable request.

**Ethics approval and consent to participate**

Not applicable.

**Consent for publication**

Not applicable.

**Competing interests**

The author declares that they have no competing interests.

**Author contributions**

RSM and FC proposed and developed the scoping review protocol topic. Ideas were developed further with academic supervisors NB and ED. The academic supervisors read and approved the final manuscript.

**References:**

1. National Health Service (NHS). Brain Tumours NHS website: NHS; 2020 [Description of brain tumours and grades]. Available from: https://www.nhs.uk/conditions/brain-tumours/.
2. Cancer Research UK. Survival Cancer Research UK online2019 [Brain tumour survival prognosis indicators]. Available from: https://www.cancerresearchuk.org/about-cancer/brain-tumours/survival.
3. Röttgering JG, Douw L, de Witt Hamer PC, Kouwenhoven MCM, Würdinger T, van de Ven PM, et al. Reducing severe fatigue in patients with diffuse glioma: a study protocol for an RCT on the effect of blended cognitive behavioural therapy. Trials. 2022;23(1):568.



For peer review only - http://bmjopen.bmj.com/site/about/guidelines.xhtml

4. Vogelzang NJ, Breitbart W, Cella D, Curt GA, Groopman JE, Horning SJ, et al. Patient, caregiver, and oncologist perceptions of cancer-related fatigue: results of a tripart assessment survey. The Fatigue Coalition. Semin Hematol. 1997;34(3 Suppl 2):4-12.
5. Berger AM, Mooney K, Alvarez-Perez A, Breitbart WS, Carpenter KM, Cella D, et al. Cancer-Related Fatigue, Version 2.2015. Journal of the National Comprehensive Cancer Network J Natl Compr Canc Netw. 2015;13(8):1012-39.
6. Armstrong TS, Gilbert MR. Practical strategies for management of fatigue and sleep disorders in people with brain tumors. Neuro Oncol. 2012;14 Suppl 4(Suppl 4):iv65-72.
7. Morrow GR, Roscoe JA, Kaufman ME, Bole C, Figueroa-Moseley C, Hofman M, et al. Cancer-Related Fatigue as a Late Effect. In: Rubin P, Constine LS, Marks LB, Okunieff P, editors. Late Effects of Cancer Treatment on Normal Tissues. Berlin, Heidelberg: Springer Berlin Heidelberg; 2008. p. 91-8.
8. Brown PD, Decker PA, Rummans TA, Clark MM, Frost MH, Ballman KV, et al. A Prospective Study of Quality of Life in Adults With Newly Diagnosed High-Grade Gliomas: Comparison of Patient and Caregiver Ratings of Quality of Life. American Journal of Clinical Oncology. 2008;31(2).
9. Asher A, Fu JB, Bailey C, Hughes JK. Fatigue among patients with brain tumors. CNS Oncol. 2016;5(2):91-100.
10. Silvaggi F, Leonardi M, Raggi A, Eigenmann M, Mariniello A, Silvani A, et al. Employment and Work Ability of Persons With Brain Tumors: A Systematic Review. Front Hum Neurosci. 2020;14:571191.
11. van Coevorden‐van Loon EMP, Heijenbrok‐Kal MH, Horemans HLD, Boere R, de Bat RAKM, Vincent AJPE, et al. The relationship between mental fatigue, cognitive functioning, and employment status in patients with low-grade glioma: a cross-sectional single-center study. Disability and Rehabilitation. 2021:1-7.
12. Ninot G. Defining Non-pharmacological Interventions (NPIs). In: Ninot G, editor. Non-Pharmacological Interventions: An Essential Answer to Current Demographic, Health, and Environmental Transitions. Cham: Springer International Publishing; 2021. p. 1-46.
13. World Health Organization WHO. Adherence to long-term therapies: evidence for action: World Health Organization; 2003.
14. Lee JK, Grace KA, Taylor AJ. Effect of a Pharmacy Care Program on Medication Adherence and Persistence, Blood Pressure, and Low-Density Lipoprotein CholesterolA Randomized Controlled Trial. JAMA. 2006;296(21):2563-71.
15. Day J, Yust-Katz S, Cachia D, Wefel J, Tremont Lukats IW, Bulbeck H, et al. Interventions for the management of fatigue in adults with a primary brain tumour. Cochrane Database of Systematic Reviews. 2022(9).
16. Boele FW, Douw L, de Groot M, van Thuijl HF, Cleijne W, Heimans JJ, et al. The effect of modafinil on fatigue, cognitive functioning, and mood in primary brain tumor patients: a multicenter randomized controlled trial. Neuro Oncol. 2013;15(10):1420-8.
17. Laigle-Donadey F, Ducray F, Boone M, Diallo MH, Hajage D, Ramirez C, et al. A phase III double-blind placebo-controlled randomized study of dexamphetamine sulfate for fatigue in primary brain tumors patients: An ANOCEF trial (DXA). Neuro-Oncology Advances. 2019;1(1):vdz043.




For peer review only - http://bmjopen.bmj.com/site/about/guidelines.xhtml

18. Porter AB, Liu H, Kohli S, Cerhan JL, Sloan J, McMurray RP, et al. Efficacy of Treatment With Armodafinil for Cancer-Related Fatigue in Patients With High-grade Glioma: A Phase 3 Randomized Clinical Trial. JAMA Oncol. 2022;8(2):259-67.
19. Posadzki, P.., Pieper, D.., Bajpai, R.., Makaruk, H.., Könsgen, N.., Neuhaus, A. L. & Semwal, M. (2020). Exercise/physical activity and health outcomes: An overview of cochrane systematic reviews. *BMC Public Health* , 20(1), pp. 1724.
20. Caspersen, C. J.., Powell, K. E. & Christenson, G. M. (1985). Physical activity, exercise, and physical fitness: Definitions and distinctions for health-related research. *Public Health Rep* , 100(2), pp. 126-131.
21. Sandler CX, Matsuyama M, Jones TL, Bashford J, Langbecker D, Hayes SC. Physical activity and exercise in adults diagnosed with primary brain cancer: a systematic review. Journal of Neuro-Oncology. 2021;153(1):1-14.
22. Gehring K, Stuiver MM, Visser E, Kloek C, van den Bent M, Hanse M, et al. A pilot randomized controlled trial of exercise to improve cognitive performance in patients with stable glioma: a proof of concept. Neuro-Oncology. 2020;22(1):103-15.
23. Milbury K, Li J, Weathers SP, Mallaiah S, Armstrong T, Li Y, et al. Pilot randomized, controlled trial of a dyadic yoga program for glioma patients undergoing radiotherapy and their family caregivers. Neurooncol Pract. 2019;6(4):311-20.
24. Rooney A, Hewins W, Walker A, Withington L, Mackinnon M, Robson S, et al. Innv-23. Lifestyle coaching is feasible and improves pilot outcomes in fatigued brain tumour patients: the bt-life (brain tumours, lifestyle interventions, and fatigue evaluation) multi-centre, phase ii rct. Neuro-Oncology. 2020;22(Supplement_2):ii121-ii.
25. Gielissen MF, Verhagen CA, Bleijenberg G. Cognitive behaviour therapy for fatigued cancer survivors: long-term follow-up. Br J Cancer. 2007;97(5):612-8.
26. Boonstra A, Gielissen M, van Dulmen-den Broeder E, Blijlevens N, Knoop H, Loonen J. Cognitive Behavior Therapy for Persistent Severe Fatigue in Childhood Cancer Survivors: A Pilot Study. J Pediatr Hematol Oncol. 2019;41(4):313-8.
27. Mind. (2023). Cognitive behavioural therapy [Online]. Place of publication: Mind UK. Available at: https://www.mind.org.uk/information-support/drugs-and-treatments/talking-therapy-and-counselling/cognitive-behavioural-therapy-cbt/ [Accessed: 17/07/2023 2023].
28. Tricco AC, Lillie E, Zarin W, O'Brien KK, Colquhoun H, Levac D, et al. PRISMA Extension for Scoping Reviews (PRISMA-ScR): Checklist and Explanation. Annals of Internal Medicine. 2018;169(7):467-73.
29. Arksey H, O'Malley L. Scoping studies: towards a methodological framework. International Journal of Social Research Methodology. 2005;8(1):19-32.
30. National Institute for Clinical Excellence (NICE). Guidance on Cancer Services. Improving outcomes for people with brain and other CNS tumours. London: National Institute for Clinical Excellence; 2006.
31. McGowan J, Sampson M, Salzwedel DM, Cogo E, Foerster V, Lefebvre C. PRESS Peer Review of Electronic Search Strategies: 2015 Guideline Statement. Journal of Clinical Epidemiology. 2016;75:40-6.



For peer review only - http://bmjopen.bmj.com/site/about/guidelines.xhtml

32. Suarez-Almazor ME, Belseck E, Homik J, Dorgan M, Ramos-Remus C. Identifying clinical trials in the medical literature with electronic databases: MEDLINE alone is not enough. Control Clin Trials. 2000;21(5):476-87.
33. Stevinson C, Lawlor DA. Searching multiple databases for systematic reviews: added value or diminishing returns? Complementary Therapies in Medicine. 2004;12(4):228-32.
34. Grossetta Nardini HK WL. The Yale MeSH Analyzer [Internet]. New Haven, CT: Cushing/Whitney Medical Library; 2023 [Available from: http://mesh.med.yale.edu/.
35. Higgins JPT, Altman DG, Gøtzsche PC, Jüni P, Moher D, Oxman AD, et al. The Cochrane Collaboration's tool for assessing risk of bias in randomised trials. BMJ. 2011;343:d5928.
36. Endnote. 20 ed. Philadelphia, PA: Clarivate; 2013.
37. Veritas. Covidence systematic review software. Melbourne, Australia: Veritas Health Innovation; 2022.
38. Pace R, Pluye P, Bartlett G, Macaulay AC, Salsberg J, Jagosh J, et al. Testing the reliability and efficiency of the pilot Mixed Methods Appraisal Tool (MMAT) for systematic mixed studies review. International Journal of Nursing Studies. 2012;49(1):47-53.
39. Hong QN, Fàbregues S, Bartlett G, Boardman F, Cargo M, Dagenais P, et al. The Mixed Methods Appraisal Tool (MMAT) version 2018 for information professionals and researchers. Education for Information. 2018;34:1-7.

**Tables:**
Table 1: Inclusion and exclusion criteria for studies

**Supplementary Information:**
Supplementary Information 1: Data charting form
Supplementary Information 2: Scoping Review Search Strategy overview
Supplementary Information 3: MEDLINE (Ovid) search strategy
Supplementary Information 4: EMBASE (Ovid) search strategy
Supplementary Information 5: CENTRAL (*The Cochrane Library*) search strategy
Supplementary Information 6: CINAHL (EBSCO*Host*) search strategy
Supplementary Information 7: SCOPUS search strategy
Supplementary Information 8: Psych info (EBSCO*host*) search strategy
Supplementary Information 9: AMED (EBSCO*host*) search strategy
Supplementary Information 10: ClinicalTrials.gov search strategy

For peer review only

# Supplementary Information

Supplementary Information 1: Data charting Form

| | Study number | 1 | 2 | 3 | 4 |
|---|---|---|---|---|---|
| Study information | Author | | | | |
| | Title | | | | |
| | Publication year | | | | |
| | Study method | | | | |
| | Sample size (participants, arms, control) | | | | |
| | Study location | | | | |
| | Main conclusion | | | | |
| Participant demographics | Age (mean, standard deviation, arm ages) | | | | |
| | Gender | | | | |
| | Population description (ethnicity, socio demographic) | | | | |
| | Tumour type and grade | | | | |
| | Comorbidities | | | | |
| Study findings | Study Duration (intervention length &timepoints/ results) | | | | |
| | Intervention type | | | | |
| | Primary outcome | | | | |
| | baseline data outcomes | | | | |
| | Study findings | | | | |
| | secondary outcomes | | | | |
| | follow up data timepoints | | | | |
| | Author recommendations | | | | |
| | Personal reflections | | | | |



For peer review only - http://bmjopen.bmj.com/site/about/guidelines.xhtml

**Supplementary Information 2: Scoping Review Search Strategy overview**

| **MesH** | **Concept 1: Brain tumor** *(Primary/non-metastatic)* | **Concept 2: Fatigue** | **Concept 3: Non-Pharmacological** | **Concept 4: Interventions** | **Concept 5: Quality of life** |
|---|---|---|---|---|---|
| Mesh Key search terms | Brain cancer, glioma, tumor, tumor, neuro oncology, neoplasm, lump, anaplastic astrocytoma, glioblastoma, Medulloblastomas, Ependymomas, Oligodendrogliomas | Fatigue, cancer related fatigue, tiredness, exhaustion, weariness, lethargy, lassitude, drained, phasic alertness, executive function, sleep | Non-pharmacological, holistic, non-medical, unmedicated, non-pharmaceutical | Treatment, program, intervention, therapy, therapeutics, care, ministrations, psychosocial, exercise, physical activity, Cognitive, rehabilitation | Welfare, wellbeing, happiness, quality of life, standard of living, psycho-oncology, survivorship, mental health |
| **Data bases** *Searching electronic databases, conferences and grey literature.* | • MEDLINE, EMBASE, CINAHL, CENTRAL, SCOPUS, Psych info, AMED.<br>• MEDLINE – IN PROCESS and Other Non-Indexed Citations Via OVID.<br>• Relevant conference proceedings will be searched by the lead author using the OVID, PROSPERO, The Cochrane Library, SCOPUS and the International Clinical Trials Registry platforms.<br>• Grey literature database ETHOS will also be searched. | | | | |
| **Inclusion criteria** | All primary research designs<br>Published from year 2006 onwards (NICE guidelines MD update)<br>Participants: Patients with a PBT<br>Intervention (non-pharmacological)<br>English Language<br>Over 18 (adult) | | | | |
| **Exclusion criteria** | Animal studies<br>Children (Persons under18 years)<br>Editorial or commentaries<br>Secondary data | | | | |
| **Time frame** | 2006- 2023. | | | | |



For peer review only - http://bmjopen.bmj.com/site/about/guidelines.xhtml

**Supplementary Information 3: MEDLINE (Ovid) search strategy**

1. exp brain neoplasms/
2. exp glioma/
3. exp neoplasms/
4. brain cancer.mp. or glioma.mp. or tumor.mp. or neurooncology.mp. or neoplasm.mp. or lump.mp. or anaplastic astrocytoma.mp. or glioblastoma.mp. or medulloblastoma.mp. or ependymoma.mp. or oligodendroglioma.mp.
5. 1 or 2 or 3 or 4
6. exp fatigue/
7. (cancer related fatigue or tiredness or exhaustion or weariness or lethargy or lassitude or drained or phasic alertness or executive function or sleep).mp.
8. 6 or 7
9. exp nonpharmacologic therapy/
10. (nonpharmacologic or holistic or nonmedical or unmedicated or nonpharmaceutical).mp.
11. 9 or 10
12. exp treatment outcome/
13. (treatment or program or intervention or therapy or therapeutics or care or ministrations or psychosocial or exercise or physical activity or rehabilitation or cognitive).mp.
14. 12 or 13
15. exp quality of life/
16. (welfare or wellbeing or happiness or quality of life or standard of living or psychooncology or survivorship or mental health).mp.
17. 15 or 16
18. 5 or 8 or 11 or 14 or 17
19. animals not humans.sh.
20. exp child/
21. 19 not 20
22. limit 21 to english language
23. limit 22 to ("2006/01/01"-"current time")

*Exp = exploded indexing term, mp = Multifield plus, sh = Subject heading.*

For peer review only

**Supplementary Information 4: EMBASE (Ovid) search strategy**

1. Exp Brain Neoplasms/
2. Exp Glioma/
3. Exp Neoplasms/
4. (Brain cancer OR Glioma OR tumor OR neuro-oncology OR neoplasm OR lump OR anaplastic astrocytoma OR glioblastoma OR medulloblastoma OR ependymoma OR oligodendroglioma).tw.
5. Neurooncology/
6. 1 or 2 or 3 or 4 or 5
7. Exp Fatigue/



For peer review only - http://bmjopen.bmj.com/site/about/guidelines.xhtml

8. (Cancer related fatigue OR tiredness OR exhaustion OR weariness OR lethargy OR lassitude OR drained OR phasic alertness OR executive function OR sleep).tw.
9. Exp non-pharmacological therapy/
10. (non-pharmacological OR holistic OR non-medical OR unmedicated OR non-pharmaceutical).tw.
11. Exp Treatment/
12. (program OR intervention OR therapy OR therapeutics OR care OR ministrations OR psychosocial OR exercise OR physical activity OR rehabilitation OR cognitive).tw.
13. Exp Quality of Life/
14. (welfare OR wellbeing OR happiness OR quality of life OR standard of living OR psycho-oncology OR survivorship OR mental health).tw.
15. 6 or 8 or 10 or 12 or 14
16. Exp Animals/ not human/
17. Exp Child/
18. 16 not 17
19. Limit 18 to English language
20. Limit 19 to 2006-current

*Exp = exploded indexing term, tw = text word search in title or abstract fields.*

**Supplementary Information 5: CENTRAL (*The Cochrane Library*) search strategy**

1. MeSH descriptor: [Brain Neoplasms] explode all trees
2. MeSH descriptor: [Glioma] explode all trees
3. MeSH descriptor: [Neoplasms]
4. Brain cancer OR glioma OR tumor OR neuro-oncology OR neoplasm OR lump OR anaplastic astrocytoma OR glioblastoma OR medulloblastoma OR ependymoma OR oligodendroglioma
5. MeSH descriptor: [Neuro-oncology] explode all trees
6. 1 or 2 or 3 or 4 or 5
7. MeSH descriptor: [Fatigue] explode all trees
8. Cancer-related fatigue or tiredness or exhaustion or weariness or lethargy or lassitude or drained or phasic alertness or executive function or sleep
9. 7 or 8
10. MeSH descriptor: [Complementary Therapies] explode all trees
11. Non-pharmacological or holistic or non-medical or unmedicated or non-pharmaceutical
12. 10 or 11
13. MeSH descriptor: [Therapeutics] explode all trees
14. Treatment or program or intervention or therapy or therapeutics or care or ministrations or psychosocial or exercise or physical activity or rehabilitation or cognitive



For peer review only - http://bmjopen.bmj.com/site/about/guidelines.xhtml

15. 13 or 14
16. MeSH descriptor: [Quality of Life] explode all trees
17. Welfare or wellbeing or happiness or quality of life or standard of living or psycho-oncology or survivorship or mental health
18. 16 or 17
19. 6 or 9 or 12 or 15 or 18
20. MeSH descriptor: [Animals] explode all trees, except humans
21. MeSH descriptor: [Child] explode all trees
22. 20 or 21
23. 19 not 22
24. Limit 23 to 2006-current
25. Limit 24 to English

**Supplementary Information 6: CINAHL (EBSCO*Host*) search strategy**

1. (Brain tumor).mh
2. (Glioma).mh
3. (Neoplasms).mh
4. Brain cancer OR Glioma OR tumor OR neuro oncology OR neoplasm OR lump OR anaplastic astrocytoma OR glioblastoma OR Medulloblastoma OR Ependymoma OR Oligodendroglioma
5. MeSH descriptor: [Neurooncology] explode all trees
6. 1 or 2 or 3 or 4 or 5
7. (fatigue).mh
8. Cancer related fatigue or tiredness or exhaustion or weariness or lethargy or lassitude or drained or phasic alertness or executive function or sleep
9. 7 or 8
10. (non-pharmacological).mh
11. non-pharmacological or holistic or non-medical or unmedicated or non-pharmaceutical
12. 10 or 11
13. (intervention).mh
14. Treatment or program or intervention or therapy or therapeutics or care or ministrations or psychosocial or exercise or physical activity or rehabilitation or Cognitive
15. 13 or 14
16. (quality of life).mh
17. Welfare or wellbeing or happiness or quality of life or standard of living or psycho-oncology or survivorship* or mental health
18. 6 or 9 or 12 or 15 or 17
19. animals/ not humans
20. children
21. 19 Not 20
22. Limit 21 to 2006- current



For peer review only - http://bmjopen.bmj.com/site/about/guidelines.xhtml

For peer review only

23. Limit 22 to English

*mh: Major heading, mm: Minor heading. *: truncation*

**Supplementary Information 7: SCOPUS search strategy**

1. EXP Brain tumor$
2. EXP Glioma$
3. EXP Neoplasms$
4. Brain cancer OR Glioma OR tumor OR neuro oncology OR neoplasm OR lump OR anaplastic astrocytoma OR glioblastoma OR Medulloblastoma OR Ependymoma OR Oligodendroglioma
5. EXP Neurooncology$
6. 1 OR 2 OR 3 OR 4 OR 5
7. EXP Fatigue$.TW.
8. "Cancer related fatigue" OR tiredness OR exhaustion OR weariness OR lethargy OR lassitude OR drained OR "phasic alertness" OR "executive function" OR sleep
9. 7 OR 8
10. EXP non-pharmacological$
11. non-pharmacological OR holistic OR "non-medical" OR unmedicated OR "non-pharmaceutical"
12. 10 OR 11
13. EXP intervention$
14. Treatment OR program OR intervention OR therapy OR therapeutics OR care OR ministrations OR psychosocial OR exercise OR "physical activity" OR rehabilitation OR Cognitive
15. 13 OR 14
16. EXP Quality of Life$
17. Welfare OR wellbeing OR happiness OR "quality of life" OR "standard of living" OR "psycho-oncology" OR survivorship$ OR "mental health"
18. 16 OR 17
19. 6 OR 9 OR 12 OR 15 OR 18
20. EXP animals/ NOT humans.SH.
21. EXP children#.SH.
22. 20 OR 21
23. 19 NOT 22
24. LIMIT-TO (SRCTITLE, "SCOPUS") AND PUBYEAR > 2005
25. LIMIT-TO (LANGUAGE, "English")

*$ = identify all words beginning with the stem, # = controlled vocabulary alternative spellings, Exp = exploded indexing term, TW = text word search in title or abstract fields.*



For peer review only - http://bmjopen.bmj.com/site/about/guidelines.xhtml

**Supplementary Information 8: Psych info (EBSCO*host*) search strategy**

1. "Brain tumour".sh
2. "Glioma".sh
3. "Neoplasms".sh
4. Brain cancer OR Glioma OR tumor OR neuro-oncology OR neoplasm OR lump OR anaplastic astrocytoma OR glioblastoma OR medulloblastoma OR Ependymoma OR Oligodendroglioma
5. "Neurooncology".sh
6. 1 or 2 or 3 or 4 or 5
7. "Fatigue".tw
8. Cancer related fatigue or tiredness or exhaustion or weariness or lethargy or lassitude or drained or phasic alertness or executive function or sleep
9. 7 or 8
10. "Non-pharmacological".tw
11. non-pharmacological or holistic or non-medical or unmedicated or non-pharmaceutical
12. 10 or 11
13. "Intervention".tw
14. Treatment or program or intervention or therapy or therapeutics or care or ministrations or psychosocial or exercise or physical activity or rehabilitation or Cognitive
15. 13 or 14
16. "Quality of Life".tw
17. Welfare or wellbeing or happiness or quality of life or standard of living or psycho-oncology or survivorship or mental health
18. 16 or 17
19. 6 or 9 or 12 or 15 or 18
20. "Animals".sh NOT Humans.sh
21. "Child".sh
22. 20 or 21
23. 19 NOT 22
24. Limit 23 to English
25. Limit 24 to United Kingdom
26. Limit 25 to 2006-Current

*Sh = Subject heading, tw = text word search in title or abstract fields.*

For peer review only

**Supplementary Information 9: AMED (EBSCO*host*) search strategy**

1. exp Brain Neoplasms/
2. exp Glioma/
3. exp Neoplasms/



For peer review only - http://bmjopen.bmj.com/site/about/guidelines.xhtml

4. brain cancer OR glioma OR tumor OR neuro-oncology OR neoplasm OR lump OR anaplastic astrocytoma OR glioblastoma OR medulloblastoma OR ependymoma OR oligodendroglioma
5. exp Neurooncology/
6. (1 or 2 or 3 or 4 or 5)
7. exp Fatigue/ or Fatigue.tw.
8. cancer related fatigue or tiredness or exhaustion or weariness or lethargy or lassitude or drained or phasic alertness or executive function or sleep
9. (7 or 8)
10. exp Complementary Therapies/ or exp Holistic Health/
11. non-pharmacological or holistic or non-medical or unmedicated or non-pharmaceutical
12. (10 or 11)
13. exp Treatment Outcome/ or exp Rehabilitation/ or exp Psychosocial Intervention/ or exp Exercise/ or exp Cognitive Therapy/
14. treatment or program or intervention or therapy or therapeutics or care or ministrations or psychosocial or exercise or physical activity or rehabilitation or cognitive
15. (13 or 14)
16. exp Quality of Life/
17. welfare or wellbeing or happiness or quality of life or standard of living or psycho-oncology or survivorship or mental health
18. (16 or 17)
19. animals/ not humans.sh.
20. children.sh.
21. (19 not 20)
22. limit 21 to english language
23. limit 22 to "2006-Current"

*Exp = exploded indexing term, tw = text word search in title or abstract fields, Sh = Subject heading.*

For peer review only

**Supplementary Information 10: ClinicalTrials.gov search strategy**

(Brain cancer OR Glioma OR tumor OR tumor OR neuro oncology OR neoplasm OR lump OR anaplastic astrocytoma OR glioblastoma OR Medulloblastoma OR Ependymoma OR Oligodendroglioma AND (fatigue OR Cancer related fatigue OR tiredness OR exhaustion or weariness OR lethargy OR lassitude OR drained OR phasic alertness OR executive function OR sleep) AND (non-pharmacological OR holistic OR non-medical OR unmedicated OR non-pharmaceutical) AND (Intervention OR Treatment OR program OR intervention OR therapy OR therapeutics OR care or ministrations OR psychosocial OR exercise OR physical activity OR rehabilitation OR Cognitive) AND (Quality of Life OR Welfare OR wellbeing OR happiness OR quality of life OR standard of living OR psycho-oncology OR survivorship OR mental health)

*Boolean search terms: AND, OR, NOT*



For peer review only - http://bmjopen.bmj.com/site/about/guidelines.xhtml

Preferred Reporting Items for Systematic reviews and Meta-Analyses extension for Scoping Reviews (PRISMA-ScR) Checklist (2018).

| SECTION | ITEM | PRISMA-ScR CHECKLIST ITEM | REPORTED ON PAGE # |
|---|---|---|---|
| **TITLE** | | | |
| Title | 1 | Identify the report as a scoping review. | 1 |
| **ABSTRACT** | | | |
| Structured summary | 2 | Provide a structured summary that includes (as applicable): background, objectives, eligibility criteria, sources of evidence, charting methods, results, and conclusions that relate to the review questions and objectives. | 2 |
| **INTRODUCTION** | | | |
| Rationale | 3 | Describe the rationale for the review in the context of what is already known. Explain why the review questions/objectives lend themselves to a scoping review approach. | 3-4 |
| Objectives | 4 | Provide an explicit statement of the questions and objectives being addressed with reference to their key elements (e.g., population or participants, concepts, and context) or other relevant key elements used to conceptualize the review questions and/or objectives. | 4 |
| **METHODS** | | | |
| Protocol and registration | 5 | Indicate whether a review protocol exists; state if and where it can be accessed (e.g., a Web address); and if available, provide registration information, including the registration number. | Click here to enter text. |
| Eligibility criteria | 6 | Specify characteristics of the sources of evidence used as eligibility criteria (e.g., years considered, language, and publication status), and provide a rationale. | 4-5 |
| Information sources* | 7 | Describe all information sources in the search (e.g., databases with dates of coverage and contact with authors to identify additional sources), as well as the date the most recent search was executed. | 5 |
| Search | 8 | Present the full electronic search strategy for at least 1 database, including any limits used, such that it could be repeated. | 13-18 |
| Selection of sources of evidence† | 9 | State the process for selecting sources of evidence (i.e., screening and eligibility) included in the scoping review. | 4-5 |
| Data charting process‡ | 10 | Describe the methods of charting data from the included sources of evidence (e.g., calibrated forms or forms that have been tested by the team before their use, and whether data charting was done independently or in duplicate) and any processes for obtaining and confirming data from investigators. | 5-6 |

For peer review only

| SECTION | ITEM | PRISMA-ScR CHECKLIST ITEM | REPORTED ON PAGE # |
|---|---|---|---|
| Data items | 11 | List and define all variables for which data were sought and any assumptions and simplifications made. | 11 |
| Critical appraisal of individual sources of evidence§ | 12 | If done, provide a rationale for conducting a critical appraisal of included sources of evidence; describe the methods used and how this information was used in any data synthesis (if appropriate). | N/A |
| Synthesis of results | 13 | Describe the methods of handling and summarizing the data that were charted. | N/A |
| **RESULTS** | | | |
| Selection of sources of evidence | 14 | Give numbers of sources of evidence screened, assessed for eligibility, and included in the review, with reasons for exclusions at each stage, ideally using a flow diagram. | N/A |
| Characteristics of sources of evidence | 15 | For each source of evidence, present characteristics for which data were charted and provide the citations. | N/A |
| Critical appraisal within sources of evidence | 16 | If done, present data on critical appraisal of included sources of evidence (see item 12). | N/A |
| Results of individual sources of evidence | 17 | For each included source of evidence, present the relevant data that were charted that relate to the review questions and objectives. | N/A |
| Synthesis of results | 18 | Summarize and/or present the charting results as they relate to the review questions and objectives. | N/A |
| **DISCUSSION** | | | |
| Summary of evidence | 19 | Summarize the main results (including an overview of concepts, themes, and types of evidence available), link to the review questions and objectives, and consider the relevance to key groups. | N/A |
| Limitations | 20 | Discuss the limitations of the scoping review process. | 2 |
| Conclusions | 21 | Provide a general interpretation of the results with respect to the review questions and objectives, as well as potential implications and/or next steps. | 6 |
| **FUNDING** | | | |
| Funding | 22 | Describe sources of funding for the included sources of evidence, as well as sources of funding for the scoping review. Describe the role of the funders of the scoping review. | 7 |

For peer review only

BMJ Open

# Non-pharmacological fatigue interventions for patients with a primary brain tumour: a scoping review protocol.



| | |
|---|---|
| Journal: | *BMJ Open* |
| Manuscript ID | bmjopen-2023-078183.R1 |
| Article Type: | Protocol |
| Date Submitted by the Author: | 08-Nov-2023 |
| Complete List of Authors: | Simms-Moore, Rachael; University of the West of England, School of Health and Social Wellbeing<br>Dures, Emma; University of the West of England, Rheumatology and Self-Management<br>Barua, Neil; Southmead Hospital, Neurosurgery<br>Cramp, Fiona; University of the West of England, College of Health, Science & Society |
| <b>Primary Subject Heading</b>: | Neurology |
| Secondary Subject Heading: | Oncology, Public health |
| Keywords: | Fatigue, Head & neck tumours < ONCOLOGY, PUBLIC HEALTH |
| | |



SCHOLARONE™
Manuscripts

**TITLE** Non-pharmacological fatigue interventions for patients with a primary brain tumour: a scoping review protocol.

**Word count**: 3,431 *(excluding references)*

Rachael Simms-Moore[1], Dr Emma Dures[2], Dr Neil Barua[3], Professor Fiona Cramp[4].

[1]Rachael Simms-Moore, University of the West of England, Bristol, rachael.simms-moore@uwe.ac.uk
[2]Dr Emma Dures, University of the West of England, Bristol, Emma2.Dures@uwe.ac.uk
[3]Dr Neil Barua, North Bristol NHS Trust, neilbarua@doctors.org.uk
[4]Professor Fiona Cramp, University of the West of England, Bristol, Fiona.Cramp@uwe.ac.uk

**Corresponding author:**
Rachael Simms-Moore[1]

Postal address:
School of Health and Social Wellbeing
College of Health, Science and Society
UWE Bristol
Glenside Campus
Blackberry Hill
BS16 1DD

Email address: rachael.simms-moore@uwe.ac.uk

Telephone: 07534389777

For peer review only



For peer review only - http://bmjopen.bmj.com/site/about/guidelines.xhtml

## ABSTRACT

### Introduction

Fatigue is the most prevalent symptom for patients with a primary brain tumour. Significantly reducing quality of life and limiting daily activities. Currently, there are limited options for managing cancer related fatigue in patients with a primary brain tumour, using non-pharmacological methods. The objective of this scoping review is to identify current and emerging evidence in relation to non-pharmacological cancer related fatigue interventions for patients with a primary brain tumour.

### Methods and analysis

Electronic databases OVID and EBSCO platforms: MEDLINE, EMBASE, and CINAHL will be searched. In addition, PROSPERO, The Cochrane Library, and ISI Web of Science will be searched. Trials registries CENTRAL and the International Clinical Trials Registry platform will also be searched for ongoing research. Inclusion criteria: studies from 2006 onwards, primary research on non-pharmacological interventions in patients with a primary brain tumour (>18 years). A PRISMA flow diagram to summarise the screening process and results.

Quantitative data will be analysed descriptively, while content analysis will be used for qualitative data.

Findings will map the existing and emerging evidence on non-pharmacological interventions for cancer-related fatigue in patients with primary brain tumours.

This will provide insights into the extent and nature of the evidence in this evolving field, identifying gaps in knowledge and research priorities, and guide further investigations in this area.

### Ethics and dissemination

Ethical approval is not required for this scoping review. Findings will be disseminated via relevant peer-reviewed journals, PhD thesis, conference presentations, and shared with relevant charities and health professionals.


**Keywords:** primary brain tumour, fatigue, adult, cancer related fatigue, non-pharmacological

## ARTICLE SUMMARY

### Strengths and limitations of this study

- Using the PRISMA- Extension for Scoping Reviews framework ensures replicability and transparency.
- Two independent researchers will assess the included studies, enhancing the reliability of the review.
- Inclusion of studies from 2006 onwards captures recent and relevant research.
- Broad evidence capture addresses an under-researched problem in a niche patient population.
- Potential bias due to the exclusion of studies not available in English.



For peer review only - http://bmjopen.bmj.com/site/about/guidelines.xhtml

**INTRODUCTION**

A primary brain tumour (PBT) is a growth of cells originating in the brain that multiplies in "an abnormal, uncontrollable way" [1]. PBT's can be categorised into high grade (grades 3-4) and lower grade (grade 1-2) tumours, with high-grade PBTs exhibiting more rapid growth than lower-grade PBT's [1]. Gliomas are the most common type of PBT, arising from glial cells in the brain and accounting for over half of PBT's [2].

Fatigue is the most prevalent symptom for patients with PBT [3]. Fatigue associated with having cancer and consequent treatments such as radiotherapy and chemotherapy is known as Cancer-Related Fatigue (CRF) [4]. The National Comprehensive Cancer Network (NCCN) defines CRF as "a distressing, persistent, subjective sense of physical, emotional, and/or cognitive tiredness or exhaustion related to cancer or cancer treatment that is not proportional to recent activity and interferes with usual functioning" [5]. CRF can involve mental, physical, and/or psychological components, with variability between patients and over time [6]. Along with experiencing unusually elevated levels of fatigue disproportionate to activity, CRF cannot be mitigated through sleep or rest [7]. CRF can significantly reduce cancer patients' quality of life [8], limiting their ability to work, continue treatment, socialise, or engage in daily activities [9-11]. Indeed, a study found that patients felt that CRF negatively impacted daily life more than pain [4].

PBTs pose distinct challenges in the management of CRF due to their direct impact on brain functions that regulate energy, sleep, mood, and cognition [12]. This complexity is exacerbated by the diverse manifestations of fatigue, which arise not only from the tumour's metabolic demands and its interference with neural pathways but also from the array of treatments such as surgery, chemotherapy, and radiation [12]. The potential use of neuro-epileptics, which are commonly prescribed for PBT-induced seizures but are rare in the treatment of other cancer types can also contribute to fatigue [13]. The trajectory of PBTs can vary significantly; high-grade tumours may progress rapidly, demanding immediate management of symptoms like CRF, while low-grade tumours may evolve more slowly, allowing for a more measured approach [1]. This suggests a need for tailoring of support to the patient journey.

There are two potential avenues for treating CRF, pharmacological interventions and non-pharmacological interventions. Non-pharmacological interventions are defined as interventions where pharmaceutical medications are not used as treatment [14], for example, lifestyle changes, psychosocial therapies, and physical activity.

Evidence suggests that patients do not want to take more drugs, with approximately 50% of patients with chronic illness not taking their medications as prescribed [15,16]. A Cochrane review (updated 2022), of "Interventions for the management of CRF in adults with a primary brain tumour" explored efficacy of interventions. This review included three randonised control trials (RCT) that had investigated pharmacological interventions for CRF [17]. All three medicines studied, Modafinil, Dexamfetamine sulphate and Amodofinil were psychostimulants; based upon the theory that promoting wakefulness could reduce CRF in patients with PBT. All three studies reported no significant effect of the pharmacological intervention on CRF compared with the control arm [18-20]. Currently, there are limited options for managing CRF in patients with a PBT [17]. Non-pharmacological evidence to date has predominantly focussed upon movement interventions, physical activity and exercise. Although the terms have been used interchangeably [21], they differ. Exercise interventions being structured, planned, repetitive activities aimed at improving fitness and health outcomes and physical activity interventions covering a broader range of movement and aim to increase overall activity levels for health and well-being [22]. In a systematic



For peer review only - http://bmjopen.bmj.com/site/about/guidelines.xhtml

review of physical activity and exercise in adults with primary brain cancer, two randomised controlled trials (RCT) reported improvements in patient fatigue [23].

One pilot RCT focused on an exercise program to improve cognitive function in patients with a PBT. It demonstrated small- to medium self-reported improvements in CRF, specifically in the areas of physical fatigue and reduced activity, with a medium effect compared to the control arm [24].

Another study by Milbury et al (2019) explored the effectiveness of dyadic yoga as an intervention for glioma patients and their families. Their study demonstrated a small clinically significant difference between the control and intervention groups, favouring the dyadic yoga intervention [25].

Overall, these findings highlight the potential of physical activity interventions in reducing CRF in patients with a PBT.

There is some limited research indicating that patients with PBT may benefit from cognitive rehabilitation programs [23] or Health Coaching programs [26]. Although not specifically studied in patients with a PBT, cognitive behavioural therapy (CBT) has been shown to reduce severe CRF in a variety of cancers [27, 28]. CBT is a talking therapy that helps treat mental health problems by focusing on how thoughts, beliefs, and attitudes impact emotions and behaviours (29). CBT teaches coping skills and challenges negative thinking patterns, aiming to improve emotional well-being (29).

Within the Cochrane review, non-pharmacological studies were organised into four main categories: educational programmes, physical activity, psychosocial/ talking therapies, and cognitive rehabilitation programs.  Overall findings indicated that there was insufficient evidence to support the effectiveness and safety of non-pharmacological treatments for CRF in patients with PBT [17]. Pharmacological interventions have demonstrated limited benefits in addressing CRF and patients are often hesitant to add more medications to their regimen. In contrast, non-pharmacological interventions have been shown to have some benefit in the management of CRF, but the RCT evidence specific to PBT is limited.  Considering primary evidence, beyond RCTs has the potential to capture a wider range of interventions, contributing to a better understanding of current knowledge in this area that could benefit patients with PBT.

**Review aims**

This review will focus on non-pharmacological interventions for managing CRF in patients with a PBT to provide a comprehensive map of published and grey literature, with the aim of highlighting knowledge gaps and identifying priority areas for future research.

## METHODS AND ANALYSIS

### Study design

Scoping reviews serve as an invaluable tool when delving into emerging research areas [30]. Unlike systematic or Cochrane reviews, which often focus on specific research designs like RCTs, scoping reviews cover a broader range of methods, ensuring a more comprehensive overview of the topic [17, 31]. This is beneficial when navigating evolving fields, such as non-pharmacological interventions for CRF in PBT patients. Our scoping review aims to bridge this gap, offering readers a comprehensive view of the current research landscape, highlighting both its strengths and areas of opportunity for further investigation.

To ensure a comprehensive and systematic approach to our scoping review, we will be employing the methodology as delineated by Arksey and O'Malley and further refined by Levac et al [31]. This methodology is widely recognised for its structured and transparent nature, and it encompasses the following stages:



For peer review only - http://bmjopen.bmj.com/site/about/guidelines.xhtml

Defining the Research Question: We have established a clear aim for this review, ensuring that our objectives are well-defined and aligned with the scope of our investigation.

Identification of Relevant Studies: We will implement a rigorous and comprehensive search strategy across multiple databases. This will ensure that we capture a broad spectrum of literature pertinent to our research question.

Selection of Studies: Based on our pre-established inclusion and exclusion criteria, we will systematically select studies that align with the objectives of our review.

Data Charting: We will extract essential details and findings from each selected study. This step will be carried out with consistency to ensure uniformity in the data collection process.

Analysis and Reporting: Once the data is charted, we will analyse it to discern patterns, emerging themes, and any evident gaps in the literature. This will provide a holistic view of the current research landscape.

Stakeholder Consultation: While this step is optional, we are considering engaging with relevant stakeholders. Their insights and perspectives can offer additional depth to our findings and ensure that our review remains relevant to the broader community.

By adhering to this structured methodology, we aim to produce a scoping review that offers a comprehensive, transparent, and insightful overview of the existing research on our topic. This review will utilise the Preferred Reporting Items for Systematic Reviews and Meta-Analyses (PRISMA) Extension for Scoping Reviews (PRISMA-ScR) framework (see Supplementary Information, research checklist) [30].

**Study question**

The review will address the question: What interventions have addressed the management of CRF in patients with PBT? The review aims to map the evidence, identify the range, recognise gaps and highlight emerging trends. By understanding the current landscape, the review will inform future research priorities.

**Identifying relevant studies**

The scoping review will include all primary research designs published from 2006 onwards. This date corresponds with the National Institute for Health and Clinical Excellence (NICE) introducing updated guidance "Improving Outcomes for People with Brain and Other CNS Tumours" [32]. This guidance marked a significant shift in the approach to care for PBT patients; promoting multidisciplinary care throughout the cancer journey to reflect fluctuating need [33]. The NICE guidance is likely to have highlighted a gap in research evidence for the management of CRF in patients with PBT, although the first RCT specific to this was not published until 2013 [18]. Commencing the scoping review search prior to publication of the updated NICE guidance is not likely to identify relevant studies.

A search strategy has been developed with input from a university-based specialist subject librarian and modified for each database (see Supplementary Information 1-8). The Peer Review of Electronic Search Strategy checklist will be used to validate the search strategy [34].

To ensure a comprehensive literature search, a range of generalised and specialised electronic databases will be searched [35, 36]. Details of the keyword searches are presented in



For peer review only - http://bmjopen.bmj.com/site/about/guidelines.xhtml

supplementary information 1-8. The following electronic databases will be searched using the OVID and EBSCO platforms: MEDLINE, EMBASE, and CINAHL. In addition, PROSPERO, The Cochrane Library, and ISI Web of Science will be searched. Trials registries CENTRAL and the International Clinical Trials Registry platform will also be searched for ongoing research.

The databases will be searched using medical subject headings (MeSH) and key search terms (see Supplementary Information 9). Keywords for each subject heading were developed using a mix of the Yale MeSH analyser software [36], free-text terms and explode command in MEDLINE, thesaurus recommendations and preferred vocabulary across databases.

In-process research such as MEDLINE – IN PROCESS and Other Non-Indexed Citations will be searched via OVID to minimise publication bias. Grey literature database Ethos will also be searched to minimise bias as positive findings are more likely to be published within peer reviewed journals [37]. A PICO model demonstrates the concept and context of this search strategy (see Supplementary Information 10).The lead author (RSM) will upload all studies and articles into referencing software EndNote-20 [38] and utilise scoping review software Covidence (Veritas Health Innovation, Melbourne, Australia) to store the data Any duplicates will be removed at this stage. To identify additional relevant studies that might have been missed in the initial database search the reference lists of included papers will be searched. For gray literature, we will employ the de-duplication strategy as suggested by Bramer et al. (2016) [39].

**Selection of studies**

Studies found will then be screened using inclusion and exclusion criteria (Table 1).

Table 1: Inclusion and exclusion criteria for studies

| Inclusion | Exclusion |
|---|---|
| • Any primary research designs<br>• Published from year 2006 onwards<br>• Available in English language<br>• Patients with a PBT ≥18 years<br>• Includes separate data for adults with a PBT<br>• PBT Glioma grades 2-4<br>• Studies including mixed non-pharmacological interventions<br>• Non-pharmacological intervention being investigated for its effect on CRF | • Animal studies<br>• Children (Persons under 18 years)<br>• Editorial or commentaries<br>• Secondary data such as systematic reviews<br>• Studies including participants with grade 1 or metastatic brain tumours<br>• Childhood brain tumours in patients who are now adults |

In this study PBT's of grade 2-4 will be included. This is supported by previous research by Röttgering (2022), finding that there was not a significant difference in fatigue severity reported by individuals with different tumour grades ranging from 2-4 [3].

To ensure a holistic representation of the non-pharmacological strategies employed for PBT patients, it is imperative to encompass studies that combine multiple interventions. This approach not only broadens the scope of our review but also mirrors the multifaceted care strategies often employed in clinical settings.

The screening process will begin with two independent reviewers (RSM and AB) reviewing the titles, followed by the abstracts, and any resources with doubt or disagreement will be retained. Next, both reviewers will independently screen the full texts using the inclusion and exclusion criteria. If there are any disagreements these will be discussed initially and if necessary, the director of studies (FC) will be consulted for conciliation.



For peer review only - http://bmjopen.bmj.com/site/about/guidelines.xhtml

**Data charting process**

Study characteristics, design, aims, participants, interventions, outcome measures, findings and authors conclusions will be collected into a data charting form (DCF) (see Supplementary Information 11). The DCF will initially be piloted by the lead author and first supervisor on 4-5 papers (RSM and FC) and refined as necessary. An independent reviewer (AB) will carry out data extraction on the studies for process validation.

**Analysis of data**

Data analysis is likely to be narrative with content analysis considered for qualitative data. All data will be summarised to produce a map of the current literature available.

Content Analysis will be conducted following a systematic, inductive approach [40]. First, we will perform Data Collection by extracting key information from each study using a standardised form. Next, we will complete Coding, where specific data pieces will be labelled to categorise them; for example, we might use the label "Physical Interventions" for all strategies related to physical activity. After coding, we will proceed to Themes, where we group similar codes together to identify broader themes, such as grouping various interventions under "Lifestyle Interventions." This will be followed by Refinement, where we will adjust categories to ensure clarity and accuracy in our analysis. Subsequently, we will move to Interpretation follows, where we will analyse the data to identify patterns and gaps in the literature. To present our findings effectively, we will employ Visualisation techniques, including tables or charts. Lastly, to ensure the reliability of our analysis, multiple reviewers will code some studies, and any discrepancies will be resolved for consistency. Through this comprehensive inductive content analysis approach, we aim to systematically categorise and interpret the literature, shedding light on key trends and areas that require further research.

**Assessment of methodological quality of individual studies**

Two reviewers (AB and RSM) will independently complete a quality assessment of each included study using the enhanced mixed methods appraisal tool (MMAT). The MMAT tool was selected as the review is likely to contain quantitative and qualitative studies, therefore a mixed methods quality assessment tool is appropriate [41]. The enhanced MMAT version was selected as it focuses on five core quality criteria to increase efficiency [42].

**Collating, summarising, and reporting**

Gaps will be highlighted and where appropriate recommendations for future research and clinical practice will be identified. Findings will be presented in a PRISMA flow diagram, mapping the number of resources identified and reasons for those included and excluded [30]. Included studies will be summarised in tabular format.

Further figures may be used to illustrate a map of the literature and gaps highlighted.

**Patient and public involvement**

This research has been discussed with patient research partners and representatives of two relevant brain tumour charities who agree that this is a priority for research. They will continue to work alongside the lead researcher in an advisory role and the findings from the review will be shared with them for further discussion. The findings derived from this study have the potential to inform and guide the research team in their endeavour to design a non-pharmacological intervention for managing fatigue in patients with a brain tumour.

**Discussion**

There is no universally accepted standard treatment for CRF, and a broad range of diverse non-pharmacological strategies exist for managing fatigue in patients with a PBT [17]. This scoping review aims to expand upon the findings of a previous Cochrane review (2015) and comprehensively examine the available evidence encompassing a broad range of non-pharmacological interventions for CRF in patients with PBT.

By incorporating all primary research evidence, the review seeks to provide a comprehensive overview of the extent and type of evidence available in this developing field. It aims to



For peer review only - http://bmjopen.bmj.com/site/about/guidelines.xhtml

identify gaps and priorities for future research, ultimately contributing to a better understanding of the current state of knowledge in this area. The results may identify interventions that are worthy of further research investment and provide recommendations for clinical practice. Through this exploration of non-pharmacological interventions for managing CRF in patients with PBT, we aim to enhance understanding and guide decision-making and identify potential interventions that could benefit patients with a PBT in the future.

## ETHICS AND DISSEMINATION

Ethical approval is not required for this scoping review. The findings of this review will be disseminated via relevant peer-reviewed journals, PhD thesis, conference presentations, and through sharing findings with relevant charities and health professionals.

**Amendments**

The protocol will be closely followed throughout with RSM regularly reporting to the supervisory team (FC, NB & ED). If any amendments are made to the published study protocol, these will be reported in the final publication.

**Supplementary Information**

Search strategies for databases
Scoping Review PICO model
Data charting form

**Abbreviations**

CRF- Cancer related fatigue
PBT- Primary brain tumour
RSM- Rachael Simms-Moore (Lead Author)
FC- Fiona Cramp (Director of Studies)
NB- Neil Barua (Supervisor)
ED- Emma Dures (Supervisor)
AB- Amy Belisario (independent reviewer)

**Acknowledgements**

None

**Funding**

This scoping review is funded as part of a PhD studentship by the University of the West of England Bristol and Somerset, Wiltshire, Avon and Gloucestershire (SWAG) Cancer Services held by RSM, Project 9187471. The funders had no role in this scoping review protocol.

**Availability of data and materials**

The datasets used and/or analysed during the current study are available from the corresponding author on reasonable request.

**Ethics approval and consent to participate**

Not applicable.

**Consent for publication**

Not applicable.

**Competing interests**

The author declares that they have no competing interests.

**Author contributions**

RSM and FC proposed and developed the scoping review protocol topic. Ideas were developed further with academic supervisors NB and ED. The academic supervisors read and approved the final manuscript.



For peer review only - http://bmjopen.bmj.com/site/about/guidelines.xhtml

**References:**


1. National Health Service. NHS. Description of brain tumours and grades [Internet]. 2020. [cited 2023 June 03] Available from: https://www.nhs.uk/conditions/brain-tumours/.
2. Cancer Research UK. Brain tumour survival prognosis indicators [Internet]. 2019 [cited 2023 June 03]. Available from: https://www.cancerresearchuk.org/about-cancer/brain-tumours/survival.
3. Röttgering JG, Douw L, de Witt Hamer PC, Kouwenhoven MCM, Würdinger T, van de Ven PM, et al. Reducing severe fatigue in patients with diffuse glioma: a study protocol for an RCT on the effect of blended cognitive behavioural therapy. Trials. 2022;23(1):568.
4. Vogelzang NJ, Breitbart W, Cella D, Curt GA, Groopman JE, Horning SJ, et al. Patient, caregiver, and oncologist perceptions of cancer-related fatigue: results of a tripart assessment survey. The Fatigue Coalition. Semin Hematol. 1997;34(3 Suppl 2):4-12.
5. Berger AM, Mooney K, Alvarez-Perez A, Breitbart WS, Carpenter KM, Cella D, et al. Cancer-Related Fatigue, Version 2.2015. J Natl Compr Canc Netw. 2015;13(8):1012-39.
6. Armstrong TS, Gilbert MR. Practical strategies for management of fatigue and sleep disorders in people with brain tumors. J. Neurooncol. 2012;14 Suppl 4(Suppl 4):iv65-72.
7. Morrow GR, Roscoe JA, Kaufman ME, Bole C, Figueroa-Moseley C, Hofman M, et al. Cancer-Related Fatigue as a Late Effect. In: Rubin P, Constine LS, Marks LB, Okunieff P, editors. Late Effects of Cancer Treatment on Normal Tissues. Berlin, Heidelberg: Springer Berlin Heidelberg. 2008. p. 91-8.
8. Brown PD, Decker PA, Rummans TA, Clark MM, Frost MH, Ballman KV, et al. A Prospective Study of Quality of Life in Adults With Newly Diagnosed High-Grade Gliomas: Comparison of Patient and Caregiver Ratings of Quality of Life. Am. J. Clin. Oncol. 2008;31(2).
9. Asher A, Fu JB, Bailey C, Hughes JK. Fatigue among patients with brain tumors. CNS Oncol. 2016;5(2):91-100.
10. Silvaggi F, Leonardi M, Raggi A, Eigenmann M, Mariniello A, Silvani A, et al. Employment and Work Ability of Persons With Brain Tumors: A Systematic Review. Front. Hum. Neurosci. 2020;14:571191.
11. Van Coevorden-van Loon EMP, Heijenbrok-Kal MH, Horemans HLD, Boere R, de Bat RAKM, Vincent AJPE, et al. The relationship between mental fatigue, cognitive functioning, and employment status in patients with low-grade glioma: a cross-sectional single-center study. Disabil. Rehabil. 2021:1-7.
12. Ryan JL, Carroll JK, Ryan EP, Mustian KM, Fiscella K, Morrow GR. Mechanisms of Cancer-Related Fatigue. Oncologist. 2007;12(S1):22-34.
13. Siniscalchi A, Gallelli L, Russo E, De Sarro G. A review on antiepileptic drugs-dependent fatigue: Pathophysiological mechanisms and incidence. Eur. J. Pharmacol. 2013;718(1):10-6.
14. Ninot G. Defining Non-pharmacological Interventions (NPIs). In: Ninot G, editor. Non-Pharmacological Interventions: An Essential Answer to Current Demographic, Health, and Environmental Transitions. Cham: Springer International Publishing; 2021. p. 1-46.




For peer review only - http://bmjopen.bmj.com/site/about/guidelines.xhtml

15. Kisa, Adnan & Sabaté, Eduardo & Nuño-Solinís, Roberto & Karkashian, Christine, et al. Adherence to long-term therapies: evidence for action: World Health Organization; 2003.
16. Lee JK, Grace KA, Taylor AJ. Effect of a Pharmacy Care Program on Medication Adherence and Persistence, Blood Pressure, and Low-Density Lipoprotein CholesterolA Randomized Controlled Trial. JAMA. 2006;296(21):2563-71.
17. Day J, Yust-Katz S, Cachia D, Wefel J, Tremont Lukats IW, Bulbeck H, et al. Interventions for the management of fatigue in adults with a primary brain tumour. Cochrane Database of Syst. Rev. 2022(9).
18. Boele FW, Douw L, de Groot M, van Thuijl HF, Cleijne W, Heimans JJ, et al. The effect of modafinil on fatigue, cognitive functioning, and mood in primary brain tumor patients: a multicenter randomized controlled trial. J. Neurooncol. 2013;15(10):1420-8.
19. Laigle-Donadey F, Ducray F, Boone M, Diallo MH, Hajage D, Ramirez C, et al. A phase III double-blind placebo-controlled randomized study of dexamphetamine sulfate for fatigue in primary brain tumors patients: An ANOCEF trial (DXA). J. Neurooncol. Adv. 2019;1(1):vdz043.
20. Porter AB, Liu H, Kohli S, Cerhan JL, Sloan J, McMurray RP, et al. Efficacy of Treatment With Armodafinil for Cancer-Related Fatigue in Patients With High-grade Glioma: A Phase 3 Randomized Clinical Trial. JAMA Oncol. 2022;8(2):259-67.
21. Posadzki, P.., Pieper, D.., Bajpai, R.., Makaruk, H.., Könsgen, N.., Neuhaus, A. L. & Semwal, M. (2020). Exercise/physical activity and health outcomes: An overview of cochrane systematic reviews. BMC Public Health, 20(1), pp. 1724.
22. Caspersen, C. J.., Powell, K. E. & Christenson, G. M. (1985). Physical activity, exercise, and physical fitness: Definitions and distinctions for health-related research. Public Health Rep, 100(2), pp. 126-131.
23. Sandler CX, Matsuyama M, Jones TL, Bashford J, Langbecker D, Hayes SC. Physical activity and exercise in adults diagnosed with primary brain cancer: a systematic review. J. Neurooncol. 2021;153(1):1-14.
24. Gehring K, Stuiver MM, Visser E, Kloek C, van den Bent M, Hanse M, et al. A pilot randomized controlled trial of exercise to improve cognitive performance in patients with stable glioma: a proof of concept. J. Neurooncol. 2020;22(1):103-15.
25. Milbury K, Li J, Weathers SP, Mallaiah S, Armstrong T, Li Y, et al. Pilot randomized, controlled trial of a dyadic yoga program for glioma patients undergoing radiotherapy and their family caregivers. Neurooncol Pract. 2019;6(4):311-20.
26. Rooney A, Hewins W, Walker A, Withington L, Mackinnon M, Robson S, et al. Innv-23. Lifestyle coaching is feasible and improves pilot outcomes in fatigued brain tumour patients: the bt-life (brain tumours, lifestyle interventions, and fatigue evaluation) multi-centre, phase ii rct. J. Neurooncol. 2020;22(Supplement_2):ii121-ii.
27. Gielissen MF, Verhagen CA, Bleijenberg G. Cognitive behaviour therapy for fatigued cancer survivors: long-term follow-up. Br. J. Cancer. 2007;97(5):612-8.
28. Boonstra A, Gielissen M, van Dulmen-den Broeder E, Blijlevens N, Knoop H, Loonen J. Cognitive Behavior Therapy for Persistent Severe Fatigue in Childhood Cancer Survivors: A Pilot Study. J. Pediatr. Hematol. Oncol. 2019;41(4):313-8.
29. Mind. Cognitive behavioural therapy [Internet]. 2023. [cited 2023 June 17] Available at: https://www.mind.org.uk/information-support/drugs-and-treatments/talking-therapy-and-counselling/cognitive-behavioural-therapy-cbt/



For peer review only - http://bmjopen.bmj.com/site/about/guidelines.xhtml

30. Tricco AC, Lillie E, Zarin W, O'Brien KK, Colquhoun H, Levac D, et al. PRISMA Extension for Scoping Reviews (PRISMA-ScR): Checklist and Explanation. Ann. Intern. Med. 2018;169(7):467-73.
31. Arksey H, O'Malley L. Scoping studies: towards a methodological framework. Int J Soc Res Methodol. 2005;8(1):19-32.
32. National Institute for Clinical Excellence (NICE). Guidance on Cancer Services. Improving outcomes for people with brain and other CNS tumours. London: National Institute for Clinical Excellence; 2006.
33. McGowan J, Sampson M, Salzwedel DM, Cogo E, Foerster V, Lefebvre C. PRESS Peer Review of Electronic Search Strategies: 2015 Guideline Statement. J Clin Epidemiol. 2016;75:40-6.
34. Suarez-Almazor ME, Belseck E, Homik J, Dorgan M, Ramos-Remus C. Identifying clinical trials in the medical literature with electronic databases: MEDLINE alone is not enough. Control. Clin. Trials. 2000;21(5):476-87.
35. Stevinson C, Lawlor DA. Searching multiple databases for systematic reviews: added value or diminishing returns? Complement Ther Med. 2004;12(4):228-32.
36. Grossetta Nardini HK WL. The Yale MeSH Analyzer. New Haven, CT: Cushing/Whitney Medical Library [Internet]. 2023. [cited 2023 June 17] Available from: http://mesh.med.yale.edu/.
37. Higgins JPT, Altman DG, Gøtzsche PC, Jüni P, Moher D, Oxman AD, et al. The Cochrane Collaboration’s tool for assessing risk of bias in randomised trials. BMJ-BRIT MED J. 2011;343:d5928.
38. Endnote. 20 ed. Philadelphia, PA: Clarivate; 2013.
39. Bramer, W. M.., Giustini, D.., De Jonge, G. B.., Holland, L. & Bekhuis, T. (2016). De-duplication of database search results for systematic reviews in endnote. J Med Libr Assoc. 104(3), pp. 240-243.
40. Creswell. J. W ACK, Vicki L. Plano Clark, Katherine Clegg Smith. Best Practices for Mixed Methods Research in the Health Sciences for the Office of Behavioral and Social Sciences Research; Qualitative Methods Overview Jo Moriarty. Qual. Soc. Work. 2013;12(4):541-5.
41. Pace R, Pluye P, Bartlett G, Macaulay AC, Salsberg J, Jagosh J, et al. Testing the reliability and efficiency of the pilot Mixed Methods Appraisal Tool (MMAT) for systematic mixed studies review. Int J Nurs Stud. 2012;49(1):47-53.
42. Hong QN, Fàbregues S, Bartlett G, Boardman F, Cargo M, Dagenais P, et al. The Mixed Methods Appraisal Tool (MMAT) version 2018 for information professionals and researchers. Educ. Inf. 2018;34:1-7.

**Tables:**
Table 1: Inclusion and exclusion criteria for studies

**Supplementary Information:**
Supplementary Information 1: MEDLINE (Ovid) search strategy
Supplementary Information 2: EMBASE (Ovid) search strategy
Supplementary Information 3: CENTRAL (*The Cochrane Library*) search strategy
Supplementary Information 4: CINAHL (EBSCO*Host*) search strategy
Supplementary Information 5: SCOPUS search strategy
Supplementary Information 6: Psych info (EBSCO*host*) search strategy
Supplementary Information 7: AMED (EBSCO*host*) search strategy
Supplementary Information 8: ClinicalTrials.gov search strategy
Supplementary Information 9: Scoping Review Search Strategy overview
Supplementary Information 10: Scoping Review PICO model



For peer review only - http://bmjopen.bmj.com/site/about/guidelines.xhtml

Supplementary Information 11: Data charting form

For peer review only



For peer review only - http://bmjopen.bmj.com/site/about/guidelines.xhtml

**Supplementary Information**

**Supplementary Information 1: MEDLINE (Ovid) search strategy**

1. exp brain neoplasms/
2. exp glioma/
3. exp neoplasms/
4. brain cancer.mp. or glioma.mp. or tumor.mp. or neurooncology.mp. or neoplasm.mp. or lump.mp. or anaplastic astrocytoma.mp. or glioblastoma.mp. or medulloblastoma.mp. or ependymoma.mp. or oligodendroglioma.mp.
5. 1 or 2 or 3 or 4
6. exp fatigue/
7. (cancer related fatigue or tiredness or exhaustion or weariness or lethargy or lassitude or drained or phasic alertness or executive function or sleep).mp.
8. 6 or 7
9. exp nonpharmacologic therapy/
10. (nonpharmacologic or holistic or nonmedical or unmedicated or nonpharmaceutical).mp.
11. 9 or 10
12. exp treatment outcome/
13. (treatment or program or intervention or therapy or therapeutics or care or ministrations or psychosocial or exercise or physical activity or rehabilitation or cognitive or measure or measurements or scale or education or information or instruments).mp.
14. 12 or 13
15. exp quality of life/
16. (welfare or wellbeing or happiness or quality of life or standard of living or psychooncology or survivorship or mental health).mp.
17. 15 or 16
18. 5 or 8 or 11 or 14 or 17
19. animals not humans.sh.
20. exp child/
21. 19 not 20
22. limit 21 to english language
23. limit 22 to ("2006/01/01"-"current time")

*Exp = exploded indexing term, mp = Multifield plus, sh = Subject heading.*

For peer review only

**Supplementary Information 2: EMBASE (Ovid) search strategy**

1. Exp Brain Neoplasms/
2. Exp Glioma/
3. Exp Neoplasms/
4. (Brain cancer OR Glioma OR tumor OR neuro-oncology OR neoplasm OR lump OR anaplastic astrocytoma OR glioblastoma OR medulloblastoma OR ependymoma OR oligodendroglioma).tw.
5. Neurooncology/
6. 1 or 2 or 3 or 4 or 5
7. Exp Fatigue/
8. (Cancer related fatigue OR tiredness OR exhaustion OR weariness OR lethargy OR lassitude OR drained OR phasic alertness OR executive function OR sleep).tw.
9. Exp non-pharmacological therapy/
10. (non-pharmacological OR holistic OR non-medical OR unmedicated OR non-pharmaceutical).tw.
11. Exp Treatment/
12. (program OR intervention OR therapy OR therapeutics OR care OR ministrations OR psychosocial OR exercise OR physical activity OR rehabilitation OR cognitive OR measure OR measurements OR scale OR education OR information OR instruments).tw.
13. Exp Quality of Life/
14. (welfare OR wellbeing OR happiness OR quality of life OR standard of living OR psycho-oncology OR survivorship OR mental health).tw.
15. 6 or 8 or 10 or 12 or 14
16. Exp Animals/ not human/
17. Exp Child/
18. 16 not 17
19. Limit 18 to English language
20. Limit 19 to 2006-current

*Exp = exploded indexing term, tw = text word search in title or abstract fields.*

For peer review only

**Supplementary Information 3: CENTRAL (*The Cochrane Library*) search strategy**

1. MeSH descriptor: [Brain Neoplasms] explode all trees
2. MeSH descriptor: [Glioma] explode all trees
3. MeSH descriptor: [Neoplasms]
4. Brain cancer OR glioma OR tumor OR neuro-oncology OR neoplasm OR lump OR anaplastic astrocytoma OR glioblastoma OR medulloblastoma OR ependymoma OR oligodendroglioma
5. MeSH descriptor: [Neuro-oncology] explode all trees
6. 1 or 2 or 3 or 4 or 5
7. MeSH descriptor: [Fatigue] explode all trees
8. Cancer-related fatigue or tiredness or exhaustion or weariness or lethargy or lassitude or drained or phasic alertness or executive function or sleep
9. 7 or 8
10. MeSH descriptor: [Complementary Therapies] explode all trees
11. Non-pharmacological or holistic or non-medical or unmedicated or non-pharmaceutical
12. 10 or 11
13. MeSH descriptor: [Therapeutics] explode all trees
14. Treatment or program or intervention or therapy or therapeutics or care or ministrations or psychosocial or exercise or physical activity or rehabilitation or cognitive or measure or measurements or scale or education or information or instruments
15. 13 or 14
16. MeSH descriptor: [Quality of Life] explode all trees
17. Welfare or wellbeing or happiness or quality of life or standard of living or psycho-oncology or survivorship or mental health
18. 16 or 17
19. 6 or 9 or 12 or 15 or 18
20. MeSH descriptor: [Animals] explode all trees, except humans
21. MeSH descriptor: [Child] explode all trees
22. 20 or 21
23. 19 not 22
24. Limit 23 to 2006-current
25. Limit 24 to English

For peer review only

**Supplementary Information 4: CINAHL (EBSCO*Host*) search strategy**

1. (Brain tumor).mh
2. (Glioma).mh
3. (Neoplasms).mh
4. Brain cancer OR Glioma OR tumor OR neuro oncology OR neoplasm OR lump OR anaplastic astrocytoma OR glioblastoma OR Medulloblastoma OR Ependymoma OR Oligodendroglioma
5. MeSH descriptor: [Neurooncology] explode all trees
6. 1 or 2 or 3 or 4 or 5
7. (fatigue).mh
8. Cancer related fatigue or tiredness or exhaustion or weariness or lethargy or lassitude or drained or phasic alertness or executive function or sleep
9. 7 or 8
10. (non-pharmacological).mh
11. non-pharmacological or holistic or non-medical or unmedicated or non-pharmaceutical
12. 10 or 11
13. (intervention).mh
14. Treatment or program or intervention or therapy or therapeutics or care or ministrations or psychosocial or exercise or physical activity or rehabilitation or cognitive or measure or measurements or scale or education or information or instruments
15. 13 or 14
16. (quality of life).mh
17. Welfare or wellbeing or happiness or quality of life or standard of living or psycho-oncology or survivorship* or mental health
18. 6 or 9 or 12 or 15 or 17
19. animals/ not humans
20. children
21. 19 Not 20
22. Limit 21 to 2006- current
23. Limit 22 to English

*mh: Major heading, mm: Minor heading. *: truncation*

For peer review only

**Supplementary Information 5: SCOPUS search strategy**

1. EXP Brain tumor$
2. EXP Glioma$
3. EXP Neoplasms$
4. Brain cancer OR Glioma OR tumor OR neuro oncology OR neoplasm OR lump OR anaplastic astrocytoma OR glioblastoma OR Medulloblastoma OR Ependymoma OR Oligodendroglioma
5. EXP Neurooncology$
6. 1 OR 2 OR 3 OR 4 OR 5
7. EXP Fatigue$.TW.
8. "Cancer related fatigue" OR tiredness OR exhaustion OR weariness OR lethargy OR lassitude OR drained OR "phasic alertness" OR "executive function" OR sleep
9. 7 OR 8
10. EXP non-pharmacological$
11. non-pharmacological OR holistic OR "non-medical" OR unmedicated OR "non-pharmaceutical"
12. 10 OR 11
13. EXP intervention$
14. Treatment OR program OR intervention OR therapy OR therapeutics OR care OR ministrations OR psychosocial OR exercise OR "physical activity” OR rehabilitation OR Cognitive OR measure OR measurements OR scale OR education OR information
15. 13 OR 14
16. EXP Quality of Life$
17. Welfare OR wellbeing OR happiness OR "quality of life" OR "standard of living" OR "psycho-oncology" OR survivorship$ OR "mental health"
18. 16 OR 17
19. 6 OR 9 OR 12 OR 15 OR 18
20. EXP animals/ NOT humans.SH.
21. EXP children#.SH.
22. 20 OR 21
23. 19 NOT 22
24. LIMIT-TO (SRCTITLE, "SCOPUS") AND PUBYEAR > 2005
25. LIMIT-TO (LANGUAGE, "English")

*$ = identify all words beginning with the stem, # = controlled vocabulary alternative spellings, Exp = exploded indexing term, TW = text word search in title or abstract fields.*

For peer review only

**Supplementary Information 6: Psych info (EBSCO*host*) search strategy**

1. "Brain tumour".sh
2. "Glioma".sh
3. "Neoplasms".sh
4. Brain cancer OR Glioma OR tumor OR neuro-oncology OR neoplasm OR lump OR anaplastic astrocytoma OR glioblastoma OR medulloblastoma OR Ependymoma OR Oligodendroglioma
5. "Neurooncology".sh
6. 1 or 2 or 3 or 4 or 5
7. "Fatigue".tw
8. Cancer related fatigue or tiredness or exhaustion or weariness or lethargy or lassitude or drained or phasic alertness or executive function or sleep
9. 7 or 8
10. "Non-pharmacological".tw
11. non-pharmacological or holistic or non-medical or unmedicated or non-pharmaceutical
12. 10 or 11
13. "Intervention".tw
14. Treatment or program or intervention or therapy or therapeutics or care or ministrations or psychosocial or exercise or physical activity or rehabilitation or cognitive or measure or measurements or scale or education or information or instruments
15. 13 or 14
16. "Quality of Life".tw
17. Welfare or wellbeing or happiness or quality of life or standard of living or psycho-oncology or survivorship or mental health
18. 16 or 17
19. 6 or 9 or 12 or 15 or 18
20. "Animals".sh NOT Humans.sh
21. "Child".sh
22. 20 or 21
23. 19 NOT 22
24. Limit 23 to English
25. Limit 24 to United Kingdom
26. Limit 25 to 2006-Current

*Sh = Subject heading, tw = text word search in title or abstract fields.*

For peer review only

**Supplementary Information 7: AMED (EBSCO*host*) search strategy**

1. exp Brain Neoplasms/
2. exp Glioma/
3. exp Neoplasms/
4. brain cancer OR glioma OR tumor OR neuro-oncology OR neoplasm OR lump OR anaplastic astrocytoma OR glioblastoma OR medulloblastoma OR ependymoma OR oligodendroglioma
5. exp Neurooncology/
6. (1 or 2 or 3 or 4 or 5)
7. exp Fatigue/ or Fatigue.tw.
8. cancer related fatigue or tiredness or exhaustion or weariness or lethargy or lassitude or drained or phasic alertness or executive function or sleep
9. (7 or 8)
10. exp Complementary Therapies/ or exp Holistic Health/
11. non-pharmacological or holistic or non-medical or unmedicated or non-pharmaceutical
12. (10 or 11)
13. exp Treatment Outcome/ or exp Rehabilitation/ or exp Psychosocial Intervention/ or exp Exercise/ or exp Cognitive Therapy/ or exp measure/ or exp measurements/ or exp scale/ or exp education/ or exp information/ or exp instruments
14. treatment or program or intervention or therapy or therapeutics or care or ministrations or psychosocial or exercise or physical activity or rehabilitation or cognitive
15. (13 or 14)
16. exp Quality of Life/
17. welfare or wellbeing or happiness or quality of life or standard of living or psycho-oncology or survivorship or mental health
18. (16 or 17)
19. animals/ not humans.sh.
20. children.sh.
21. (19 not 20)
22. limit 21 to english language
23. limit 22 to "2006-Current"

*Exp = exploded indexing term, tw = text word search in title or abstract fields, Sh = Subject heading.*

For peer review only

**Supplementary Information 8: ClinicalTrials.gov search strategy**

1. (Brain cancer OR Glioma OR tumor OR tumor OR neuro oncology OR neoplasm OR lump OR anaplastic astrocytoma OR glioblastoma OR Medulloblastoma OR Ependymoma OR Oligodendroglioma AND (fatigue OR Cancer related fatigue OR tiredness OR exhaustion or weariness OR lethargy OR lassitude OR drained OR phasic alertness OR executive function OR sleep) AND (non-pharmacological OR holistic OR non-medical OR unmedicated OR non-pharmaceutical) AND (Intervention OR Treatment OR program OR intervention OR therapy OR therapeutics OR care or ministrations OR psychosocial OR exercise OR physical activity OR rehabilitation OR Cognitive OR measure OR measurements OR scale OR education OR information OR) AND (Quality of Life OR Welfare OR wellbeing OR happiness OR quality of life OR standard of living OR psycho-oncology OR survivorship OR mental health)

*Boolean search terms: AND, OR, NOT*

For peer review only

**Supplementary Information 9: Scoping Review Search Strategy overview**

| **MesH** | **Concept 1: Brain tumor** *(Primary/non-metastatic)* | **Concept 2: Fatigue** | **Concept 3: Non-Pharmacological** | **Concept 4: Interventions** | **Concept 5: Quality of life** |
|---|---|---|---|---|---|
| **Mesh Key search terms** | Brain cancer, glioma, tumor, tumor, neuro oncology, neoplasm, lump, anaplastic astrocytoma, glioblastoma, Medulloblastomas, Ependymomas, Oligodendrogliomas | Fatigue, cancer related fatigue, tiredness, exhaustion, weariness, lethargy, lassitude, drained, phasic alertness, executive function, sleep | Non-pharmacological, holistic, non-medical, unmedicated, non-pharmaceutical | Treatment, program, intervention, therapy, therapeutics, care, ministrations, psychosocial, exercise, physical activity, Cognitive, rehabilitation, measure, measurements, scale, education, information, instruments | Welfare, wellbeing, happiness, quality of life, standard of living, psycho-oncology, survivorship, mental health |
| **Data bases** *Searching electronic databases, conferences and grey literature.* | • MEDLINE, EMBASE, CINAHL, CENTRAL, SCOPUS, Psych info, AMED.<br>• MEDLINE – IN PROCESS and Other Non-Indexed Citations Via OVID.<br>• Relevant conference proceedings will be searched by the lead author using the OVID, PROSPERO, The Cochrane Library, SCOPUS and the International Clinical Trials Registry platforms.<br>• Grey literature database ETHOS will also be searched. | | | | |
| **Time frame** | 2006- 2023. | | | | |

For peer review only

**Supplementary Information 10: PICO model for Scoping Review on Non-Pharmacological Interventions for Fatigue in Primary Brain Tumor Patients**

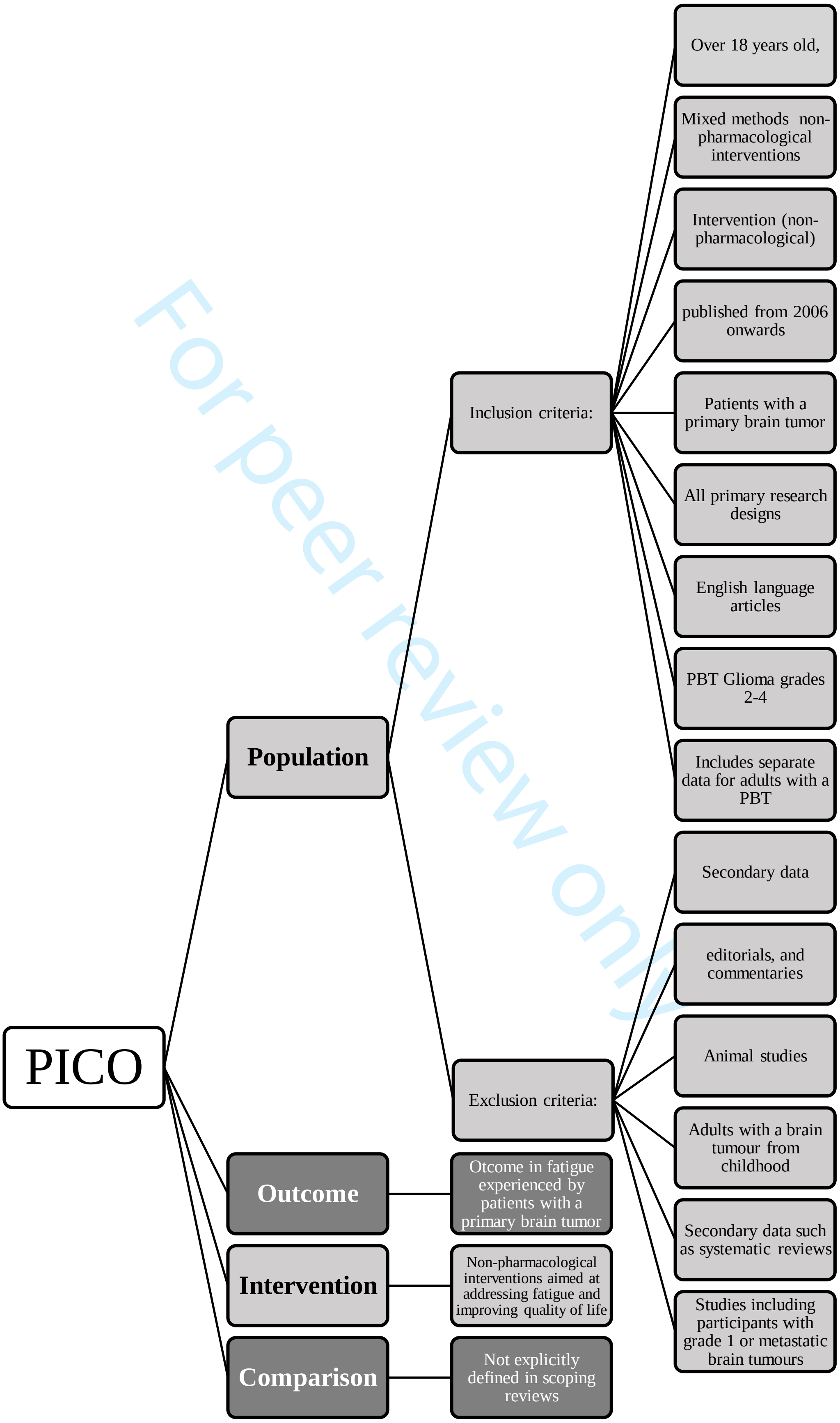

**Supplementary Information 11: Data charting Form**

| | Study number | 1 | 2 | 3 | 4 |
|---|---|---|---|---|---|
| Study information | Author | | | | |
| | Title | | | | |
| | Publication year | | | | |
| | Study method | | | | |
| | Sample size (participants, arms, control) | | | | |
| | Study location (geographic or country region) | | | | |
| | Study setting (hospital, community centre, charity center, home) | | | | |
| | Main conclusion | | | | |
| Participant demographics | Age (mean, standard deviation, arm ages) | | | | |
| | Gender | | | | |
| | Population description (ethnicity, socio demographic) | | | | |
| | Tumour type and grade | | | | |
| | Comorbidities | | | | |
| Study findings | Study Duration (intervention length &timepoints/ results) | | | | |
| | Intervention type | | | | |
| | Intervention provider (medical professionals, allied health professionals, laypersons or trained volunteers, patients' family members or caregivers, and Others- description) | | | | |
| | Intervention provider training | | | | |
| | Primary outcome | | | | |
| | baseline data outcomes | | | | |
| | Study findings | | | | |
| | secondary outcomes | | | | |
| | follow up data timepoints | | | | |
| | Author recommendations | | | | |
| | Personal reflections | | | | |

For peer review only

Preferred Reporting Items for Systematic reviews and Meta-Analyses extension for Scoping Reviews (PRISMA-ScR) Checklist (2018).

| SECTION | ITEM | PRISMA-ScR CHECKLIST ITEM | REPORTED ON PAGE # |
|---|---|---|---|
| **TITLE** | | | |
| Title | 1 | Identify the report as a scoping review. | 1 |
| **ABSTRACT** | | | |
| Structured summary | 2 | Provide a structured summary that includes (as applicable): background, objectives, eligibility criteria, sources of evidence, charting methods, results, and conclusions that relate to the review questions and objectives. | 2 |
| **INTRODUCTION** | | | |
| Rationale | 3 | Describe the rationale for the review in the context of what is already known. Explain why the review questions/objectives lend themselves to a scoping review approach. | 3-4 |
| Objectives | 4 | Provide an explicit statement of the questions and objectives being addressed with reference to their key elements (e.g., population or participants, concepts, and context) or other relevant key elements used to conceptualize the review questions and/or objectives. | 4-5 |
| **METHODS** | | | |
| Protocol and registration | 5 | Indicate whether a review protocol exists; state if and where it can be accessed (e.g., a Web address); and if available, provide registration information, including the registration number. | N/A |
| Eligibility criteria | 6 | Specify characteristics of the sources of evidence used as eligibility criteria (e.g., years considered, language, and publication status), and provide a rationale. | 5-6 |
| Information sources* | 7 | Describe all information sources in the search (e.g., databases with dates of coverage and contact with authors to identify additional sources), as well as the date the most recent search was executed. | 5-6 |
| Search | 8 | Present the full electronic search strategy for at least 1 database, including any limits used, such that it could be repeated. | Supplementary information 1-9 |
| Selection of sources of evidence† | 9 | State the process for selecting sources of evidence (i.e., screening and eligibility) included in the scoping review. | 6 |
| Data charting process‡ | 10 | Describe the methods of charting data from the included sources of evidence (e.g., calibrated forms or forms that have been tested by the team before their use, and whether data charting was done independently or in duplicate) and any processes for obtaining and confirming data from investigators. | 7 |

For peer review only

| SECTION | ITEM | PRISMA-ScR CHECKLIST ITEM | REPORTED ON PAGE # |
|---|---|---|---|
| Data items | 11 | List and define all variables for which data were sought and any assumptions and simplifications made. | Supplementary information 9 |
| Critical appraisal of individual sources of evidence§ | 12 | If done, provide a rationale for conducting a critical appraisal of included sources of evidence; describe the methods used and how this information was used in any data synthesis (if appropriate). | N/A |
| Synthesis of results | 13 | Describe the methods of handling and summarizing the data that were charted. | N/A |
| **RESULTS** | | | |
| Selection of sources of evidence | 14 | Give numbers of sources of evidence screened, assessed for eligibility, and included in the review, with reasons for exclusions at each stage, ideally using a flow diagram. | N/A |
| Characteristics of sources of evidence | 15 | For each source of evidence, present characteristics for which data were charted and provide the citations. | N/A |
| Critical appraisal within sources of evidence | 16 | If done, present data on critical appraisal of included sources of evidence (see item 12). | N/A |
| Results of individual sources of evidence | 17 | For each included source of evidence, present the relevant data that were charted that relate to the review questions and objectives. | N/A |
| Synthesis of results | 18 | Summarize and/or present the charting results as they relate to the review questions and objectives. | N/A |
| **DISCUSSION** | | | |
| Summary of evidence | 19 | Summarize the main results (including an overview of concepts, themes, and types of evidence available), link to the review questions and objectives, and consider the relevance to key groups. | N/A |
| Limitations | 20 | Discuss the limitations of the scoping review process. | 2 |
| Conclusions | 21 | Provide a general interpretation of the results with respect to the review questions and objectives, as well as potential implications and/or next steps. | 7-8 |
| **FUNDING** | | | |
| Funding | 22 | Describe sources of funding for the included sources of evidence, as well as sources of funding for the scoping review. Describe the role of the funders of the scoping review. | 8 |

For peer review only
